# Supplementary material for: Channelrhodopsin variants for high-rate optogenetic neurostimulation at low light intensities
Source: EMBO Mol Med. 2025 Dec 9;18(2):462–91. doi: 10.1038/s44321-025-00350-z (PMC12905302; doi:10.1038/s44321-025-00350-z)
Supplement: Supplementary file 1 — Table EV1 [file 44321_2025_350_MOESM1_ESM.docx]

|  | **J _-60mV_ (pA/pF)** | **τ_off_ (-60 mV)  RT** |
| --- | --- | --- |
| **Chronos (a)** | 24.01 ± 7.46 (n = 14)  # b***, c**, d(ns), e(ns), f(ns), g(ns), h(ns), i(ns) | 3.72 ± 0.67 (n = 10)  § b(ns), c(ns), d(ns), e(ns), f*, g(ns), h****, i**** |
| **f-Chronos (b)** | 4.66 ± 2.98 (n = 13)  # a***, c(ns), d(ns), e(ns), f(ns), g****, h****, i*** | 1.72 ± 0.12 ms (n = 4)  § a(ns), c(ns), d(ns), e(ns), f*, g(ns), h****, i**** |
| **f-Chronos LC (c)** | 4.55 ± 3.09 (n = 9)  # a**, b (ns), d(ns), e(ns), f(ns), g****, h****, i*** | 3.56 ± 0.94 ms (n = 8)  § a(ns), b(ns), d(ns), e(ns), f*, g(ns), h****, i**** |
| **Chronos LC (d)** | 17 ± 6.85 (n = 11)  # a(ns), b(ns), (ns), e(ns), f(ns), g(ns), h(ns), i(ns) | 8.22 ± 1.73 ms (n = 11)  § a(ns), b(ns), c(ns), e(ns), f(ns), g(ns), h**, i* |
| **ChR2 (e)** | 19.33 ± 7.57 (n = 11)  # a(ns), b(ns), c(ns), d(ns), f(ns), g(ns), h(ns), i (ns) | 10.54 ± 2.34 ms (n = 9)  § a(ns), b(ns), c(ns), d(ns), f(ns), g(ns), h(ns), i(ns) |
| **ChR2 ET/TC (f)** | 14.15 ± 5.37 (n = 11)  # a(ns), b(ns), c(ns), d(ns), e (ns), g(ns), h**, i(ns) | 10.99 ± 2.23 ms (n = 11)  § a*, b*, c*, d(ns), e(ns), g(ns), h(ns), i(ns) |
| **f-ChR2 TC (g)** | 28.42 ± 10.36 (n = 16)  # a (ns), b****, c****, d(ns), e(ns), f(ns), h(ns), i(ns) | 9.73 ± 1 ms (n = 9)  § a(ns), b(ns), c(ns), d(ns), e(ns), f(ns), h(ns), i(ns) |
| **CatCh (h)** | 37.05 ± 12.46 (n = 11)  # a(ns), b****, c****, d(ns), e(ns), f**, g(ns), i(ns) | 33.09 ± 5.72 ms (n = 9)  § a****, b****, c****, d**, e(ns), f(ns), g(ns), i (ns) |
| **ChR2 TC (i)** | 31.39 ± 17.53 (n = 10)  # a(ns), b***, c***, d(ns), e(ns), f(ns), g(ns), h(ns) | 28.22 ± 6.52 ms (n = 10)  # a****, b****, c****, d*, e(ns), f(ns), g(ns), h(ns) |

**Legend:**

**Table EV1. Stationary current densities [J_-60mV_ (pA/pF)] and closing kinetics (τ_off_ values) of blue light activated ChRs.** Stationary current densities were obtained from the quotient of the mean stationary photocurrent upon 500 ms light stimulation (saturating intensity of ~ 30 mW/mm^2^, *λ* = 473 nm) and the capacitance of the cell. ^#^ Significantly different current densities compared to a) Chronos, b) f-Chronos, c) f-Chronos LC, d) Chronos LC, e) ChR2, f) ChR2 ET/TC, g) f-ChR2 TC, h) CatCh, and i) ChR2 TC. Closing kinetics were determined at RT by a monoexponential fit of the decaying photocurrent after 3 ms light pulse (saturating intensity of ~ 30 mW/mm^2^, *λ*= 473 nm). f-Chronos closing kinetics at RT were obtained from photocurrents elicited by 7 ns light pulse (λ = 500 nm, 1020 photons/m^2^) to avoid interference in the off-kinetics due to shutter opening/closing time (~ 700 μs) using the Opolette 355 tunable laser system (Opotek Inc, Carlsbad, USA). § Significantly different closing kinetics compared to a) Chronos, b) f-Chronos, c) f-Chronos LC, d) Chronos LC, e) ChR2, f) ChR2 ET/TC, g) f-ChR2 TC, h) CatCh, and i) ChR2 TC. Kruskal-Wallis followed by Dunn’s test: p > 0.05 (ns); * p < 0.05; ** p < 0.01; *** p < 0.001; **** p < 0.0001. All measurements were performed in NG108-15 cells transiently transfected with the specified ChR variants by whole-cell patch clamp at membrane potential of -60 mV. Data are
