# Supplementary material for: Channelrhodopsin variants for high-rate optogenetic neurostimulation at low light intensities
Source: EMBO Mol Med. 2025 Dec 9;18(2):462–91. doi: 10.1038/s44321-025-00350-z (PMC12905302; doi:10.1038/s44321-025-00350-z)
Supplement: Supplementary file 2 — Table EV2 [file 44321_2025_350_MOESM2_ESM.docx]

|  | **τ_off_ (-60 mV) RT** | **τ_off_ (-60 mV) 33 to 34 °C** | **EC50 (mW/mm^2^)** | **Desensitization (Stat/Peak)** |
| --- | --- | --- | --- | --- |
| **Chronos (a)** | 3.72 ± 0.67  (n = 10) | 1.875 ± 0.47 ms  (n = 6)  § b(ns), d(ns), g(ns), h** | 1.25 ± 0.64 (n = 6)  $ b(ns), d(ns), e(ns), f(ns), g(ns), h(ns) | 0.35 ± 0.085 (n = 10)  # b****, c(ns), d****, e(ns), f(ns), g(ns), h****, i(ns) |
| **f-Chronos (b)** | 1.72 ± 0.12 ms  (n = 4) | 0.80 ± 0.099 ms  (n = 4)  § a(ns), d(ns), g*, h*** | 5.12 ± 0.91 (n = 6)  $ a(ns), d(ns), e(ns), f(ns), g(ns), h** | 0.19 ± 0.044 (n = 14)  # a****, c(ns), d****, e(ns), f*, g****, h****, i(ns) |
| **f-Chronos LC (c)** | 3.56 ± 0.94 ms  (n = 8) |  |  | 0.29 ± 0.026 (n = 3)  # a(ns), b(ns), d****, e(ns), f(ns), g(ns), h****, i(ns) |
| **Chronos LC (d)** | 8.22 ± 1.73 ms  (n = 11) | 3.93 ± 0.19 ms  (n = 4)  § a(ns), b(ns), g(ns), h(ns) | 1.02 ± 0.14 (n = 4)  $ a(ns), b(ns), e(ns), f(ns), g(ns), h(ns) | 0.62 ± 0.077 (n = 6)  # a****, b****, c****, e****, f****, g****, h(ns), i**** |
| **ChR2 (e)** | 10.54 ± 2.34 ms  (n = 9) |  | 1.29 ± 0.57 (n = 3)  $ a(ns), b(ns), d(ns), f(ns), g(ns), h(ns) | 0.26 ± 0.064 (n = 6)  # a(ns), b(ns), c(ns), d****, f(ns), g(ns), h****, i(ns) |
| **ChR2 ET/TC (f)** | 10.99 ± 2.23 ms (n = 11) |  | 1.7 ± 0.33 (n = 3)  $ a(ns), b(ns), d(ns), e(ns), g(ns), h(ns) | 0.298 ± 0.063 (n = 9)  # a(ns), b*, c(ns), d****, e(ns), g(ns), h****, i(ns) |
| **f-ChR2 TC (g)** | 9.73 ± 1 ms  (n = 9) | 4.1 ± 0.96 ms  (n = 12)  § a**, b***, d(ns), h(ns) | 1.29 ± 0.315  (n = 3)  $ a(ns), b(ns), d(ns), e(ns), f(ns), h(ns) | 0.37 ± 0.073 (n = 7)  # a(ns), b****, c(ns), d****, e(ns), f(ns), h****, i(ns) |
| **CatCh (h)** | 33.09 ± 5.72 ms  (n = 9) | 15.71 ± 2.17 ms  (n = 5)  § a**, b***, d(ns), g(ns) | 0.54 ± 0.12 (n = 3)  $ a(ns), b**, d(ns), e(ns), f(ns), g(ns) | 0.74 ± 0.058 (n = 8)  # a****, b****, c****, d(ns), e****, f****, g****, i**** |
| **ChR2 TC (i)** | 28.22 ± 6.52 ms (n = 10) |  | 0.637 (n = 1) | 0.28 ± 0.079 (n = 6)  # a(ns), b(ns), c(ns), d****, e(ns), f(ns), g(ns), i**** |

**Legend:**

**Table EV2. Closing kinetics (τ_off_ values), EC50 values and stationary-peak-ratios of blue light activated ChRs.** Closing kinetics were determined at RT by a monoexponential fit of the decaying photocurrent after 3 ms light pulse (saturating intensity of 30 to 40 mW/mm^2^, *λ* = 473 nm), and f-Chronos closing kinetics were obtained from photocurrents elicited by 7 ns light pulse (λ = 500 nm, 1,020 photons/m^2^), or more physiological temperature (33 to 34 °C; 1 ms, saturating intensity of 30 to 40 mW/mm^2^, *λ* = 488 nm). The half maximal activation value (effective power density for 50 % activation, analogous to an EC_50_) was determined by hyperbolic fitting of the stationary photocurrent amplitude obtained upon 0.5 or 1 s light pulses at different irradiances (mW/mm^2^). Desensitization values were determined by the quotient of the mean stationary photocurrent of 0.5 to 1 s light pulse and their respective peak photocurrent. All measurements were performed in NG108-15 cells transiently transfected with the specified ChR variants by whole-cell patch clamp at membrane potential of -60 mV. Data are presented as mean ± SD. All values are derived from the data shown in Figure 1. § Significantly different closing kinetics compared to a) Chronos, b) f-Chronos, d) Chronos LC, g) f-ChR2 TC, and h) CatCh. $ Significantly different EC50 compared to a) Chronos, b) f-Chronos, d) Chronos LC, e) ChR2, f) ChR2 ET/TC, g) f-ChR2 TC, and h) CatCh. ^#^ Significantly different desensitization to a) Chronos, b) f-Chronos, c) f-Chronos LC, d) Chronos LC, e) ChR2, f) ChR2 ET/TC, g) f-ChR2 TC, h) CatCh, and i) ChR2 TC. τoff (-60 mV; at 33 to 34 °C) and EC50 were tested by Kruskal-Wallis t-test and post-hoc Dunn’s test: p > 0.05 (ns); * p < 0.05; ** p < 0.01; *** p < 0.001; **** p < 0.0001. Desensitization was tested by ANOVA Bonferroni: p
