# Supplementary material for: Channelrhodopsin variants for high-rate optogenetic neurostimulation at low light intensities
Source: EMBO Mol Med. 2025 Dec 9;18(2):462–91. doi: 10.1038/s44321-025-00350-z (PMC12905302; doi:10.1038/s44321-025-00350-z)
Supplement: Supplementary file 3 — Table EV3 [file 44321_2025_350_MOESM3_ESM.docx]

|  | **Frequency (Hz)** | **Stationary/peak** | **Fluctuation/peak** |
| --- | --- | --- | --- |
| **f-ChR2 TC (a)** | 5 | 0.54 ± 0.06, n = 4  # b(ns), c(ns), d** | 0.99 ± 0.002, n = 4  $ b(ns), c(ns), d(ns) |
|  | 10 | 0.58 ± 0.06, n = 4  # b(ns), c(ns), d*** | 0.99 ± 0.009, n = 4  $ b*, c(ns), d(ns) |
|  | 20 | 0.57 ± 0.07, n = 4  # b(ns), c***, d*** | 0.98 ± 0.01, n = 4  $ b**, c(ns), d(ns) |
|  | 30 | 0.54 ± 0.06, n = 4  # b*, c***, d*** | 0.98 ± 0.01, n = 4  $ b***, c(ns), d(ns) |
|  | 40 | 0.52 ± 0.05, n = 4  # b***, c****, d**** | 0.98 ± 0.01, n = 4  $ b****, c(ns), d(ns) |
|  | 50 | 0.49 ± 0.04, n = 4  # b****, c****, d**** | 0.97 ± 0.01, n = 4  $ b****, c(ns), d(ns) |
|  | 125 | 0.38 ± 0.05, n = 4  # b****, c**, d**** | 0.84 ± 0.05, n = 4  $ b****, c(ns), d(ns) |
|  | 200 | 0.37 ± 0.05, n = 4  # b****, c**, d**** | 0.68 ± 0.07, n = 4  $ b****, c**, d* |
|  | 333 | 0.36 ± 0.05, n = 4  # b****, c**, d*** | 0.46 ± 0.09, n = 4  $ b****, c**, d* |
|  | 500 | 0.38 ± 0.06, n = 4  # b****, c***, d*** | 0.27 ± 0.09, n = 4  $ b***, c**, d(ns) |
| **CatCh (b)** | 5 | 0.6 ± 0.04, n = 4  # a(ns), c(ns), d* | 0.98 ± 0.006, n = 4  $ a(ns), c(ns), d(ns) |
|  | 10 | 0.64 ± 0.02, n = 4  # a(ns), c**, d** | 0.97 ± 0.0064, n = 4  $ a*, c*, d** |
|  | 20 | 0.63 ± 0.008, n = 4  # a(ns), c****, d** | 0.89 ± 0.04, n = 4  $ a**, c**, d** |
|  | 30 | 0.64 ± 0.01, n = 4  # a*, c****, d* | 0.73 ± 0.08, n = 4  $ a***, c***, d*** |
|  | 40 | 0.66 ± 0.02, n = 4  # a***, c****, d(ns) | 0.63 ± 0.1, n = 4  $ a****, c***, d*** |
|  | 50 | 0.68 ± 0.02, n = 4  # a****, c****, d(ns) | 0.54 ± 0.12, n = 4  $ a****, c***, d*** |
|  | 125 | 0.74 ± 0.02, n = 4  # a****, c****, d*** | 0.21 ± 0.08, n = 4  $ a****, c****, d**** |
|  | 200 | 0.78 ± 0.03, n = 4  # a****, c****, d**** | 0.11 ± 0.04, n = 4  $ a****, c****, d**** |
|  | 333 | 0.79 ± 0.04, n = 4  # a****, c****, d*** | 0.05 ± 0.02, n = 4  $ a****, c****, d** |
|  | 500 | 0.82 ± 0.03, n = 4  # a****, c****, d*** | 0.02 ± 0.01, n = 4  $ a***, c****, d(ns) |
| **f-Chronos (c)** | 5 | 0.6 ± 0.009, n = 3  # a(ns), b(ns), d* | 1 ± 0.01, n = 3  $ a(ns), b(ns), d(ns) |
|  | 10 | 0.5 ± 0.02, n = 3  # a(ns), b**, d**** | 0.99 ± 0.003, n = 3  $ a(ns), b*, d(ns) |
|  | 20 | 0.37 ± 0.01, n = 3  # a***, b****, d**** | 0.98 ± 0.01, n = 3  $ a(ns), b**, d(ns) |
|  | 30 | 0.32 ± 0.01, n = 3  # a***, b****, d**** | 0.98 ± 0.01, n = 3  $ a(ns), b***, d(ns) |
|  | 40 | 0.32 ± 0.01, n = 3  # a****, b****, d**** | 0.96 ± 0.01, n = 3  $ a(ns), b***, d(ns) |
|  | 50 | 0.3 ± 0.01, n = 3  # a****, b****, d**** | 0.94 ± 0.05, n = 3  $ a(ns), b***, d(ns) |
|  | 125 | 0.25 ± 0.008, n = 3  # a**, b****, d**** | 0.89 ± 0.01, n = 3  $ a(ns), b****, d* |
|  | 200 | 0.22 ± 0.01, n = 3  # a**, b****, d**** | 0.89 ± 0.05, n = 3  $ a**, b****, d*** |
|  | 333 | 0.21 ± 0.01, n = 3  # a**, b****, d**** | 0.7 ± 0.06, n = 3  $ a**, b****, d**** |
|  | 500 | 0.18 ± 0.02, n = 3  # a***, b****, d**** | 0.52 ± 0.04, n = 3  $ a**, b****, d**** |
| **Chronos LC (d)** | 5 | 0.75 ± 0.03, n = 3  # a**, b*, c* | 1 ± 0.004, n = 3  $ a(ns), b(ns), c(ns) |
|  | 10 | 0.82 ± 0.01, n = 3  # a***, b**, c**** | 0.99 ± 0.001, n = 3  $ a(ns), b**, c(ns) |
|  | 20 | 0.78 ± 0.01, n = 3  # a***, b**, c**** | 0.99 ± 0.004, n = 3  $ a(ns), b**, c(ns) |
|  | 30 | 0.74 ± 0.01, n = 3  # a***, b*, c**** | 0.98 ± 0.0008, n = 3  $ a(ns), b***, c(ns) |
|  | 40 | 0.73 ± 0.01, n = 3  # a****, b(ns), c**** | 0.97 ± 0.005, n = 3  $ a(ns), b***, c(ns) |
|  | 50 | 0.7 ± 0.01, n = 3  # a****, b(ns), c**** | 0.96 ± 0.005, n = 3  $ a(ns), b***, c(ns) |
|  | 125 | 0.6 ± 0.01, n = 3  # a****, b***, c**** | 0.74 ± 0.02, n = 3  $ a(ns), b****, c* |
|  | 200 | 0.57 ± 0.01, n = 3  # a****, b****, c**** | 0.53 ± 0.03, n = 3  $ a*, b****, c*** |
|  | 333 | 0.57 ± 0.006, n = 3  # a***, b***, c**** | 0.29 ± 0.02, n = 3  $ a*, b**, c**** |
|  | 500 | 0.58 ± 0.01, n = 3  # a***, b***, c**** | 0.14 ± 0.01, n = 3  $ a(ns), b(ns), c**** |

**Legend:**

**Table EV3. Frequency dependencies of stationary-peak-ratios and photocurrent fluctuations at physiological temperature.** Statistical comparison of data shown in Figure EV1. Whole-cell patch-clamp measurements at ~ 34 °C in NG108-15 cells expressing ChRs 2 to 3 days after transient transfection. ChRs were light activated by 50 light pulses of 1 ms at 488 nm (39.43 mW/mm^2^) at different frequencies. Stationary photocurrent amplitudes of the different ChR variants were divided by the peak photocurrent amplitude to show how desensitization is influenced by the frequency of stimulation using short light pulses. Data are presented as mean ± SD. # Significantly different desensitization levels at the indicated frequency of light stimulation compared to a) f-ChR2 TC, b) CatCh, c) f-Chronos, and d) Chronos LC. Quantification of the photocurrent fluctuation at the stationary state (end of the pulse train) of different ChR variants normalized to the stationary current amplitude. $ Significantly different fluctuation amplitudes at the indicated frequency of light stimulation compared to a) f-ChR2 TC, b) CatCh, c) f-Chronos, and d) Chronos LC. Multiple comparison results were obtained by ANOVA Bonferroni: p > 0.05 (ns); * p < 0.05; ** p < 0.01; *** p < 0.001; **** p < 0.0001.
