# Supplementary material for: Channelrhodopsin variants for high-rate optogenetic neurostimulation at low light intensities
Source: EMBO Mol Med. 2025 Dec 9;18(2):462–91. doi: 10.1038/s44321-025-00350-z (PMC12905302; doi:10.1038/s44321-025-00350-z)
Supplement: Supplementary file 4 — Appendix [file 44321_2025_350_MOESM4_ESM.pdf]

Appendix for

Channelrhodopsin variants for high-rate optogenetic neurostimulation at low light intensities

Roos *et al.*, 2025

Appendix Figures

Appendix Figure S1. Effect of the C-terminal fusion of targeting motifs on the plasma membrane expression of f-ChR2 TC. **page 3**

Appendix Figure S2. Recovery from photocurrent desensitization. **page 4**

Appendix Figure S3. Comparison of ChR channel closing kinetics in rat hippocampal neurons (HN) and NG cells (NG). **page 5**

Appendix Figure S4. Immunohistochemical analysis of ChR expression in exemplary oABR-positive cochleae. **pages 6-7**

Appendix Figure S5. Histological and immunohistochemical analysis of cochlear paraffin-sections. **page 8**

Appendix Figure S6. Characteristic firing pattern of AVCN neurons in response to 100 ms depolarizing current injection. **page 9**

Appendix Figure S7. Classification of principal cells in the cochlear nucleus based on their spontaneous excitatory postsynaptic currents and cell morphology. **pages 10-11**

Appendix Figure S8. Spike latency increases over the train of light pulse stimulation. **page 12**

Appendix Figure S9. Bushy cells with greater light sensitivity can follow higher frequencies of stimulation. **page 13**

Appendix Figure S10. oeEPSC measurements at different light intensities. **page 14**

Appendix Figure S11. f-ChR2 TC expression in SGNs at P20 in postnatally injected mice. **page 15**

Appendix Figure S12. oABR shape and threshold for Mongolian gerbils expressing f-ChR2 TC in SGNs. **page 16**

Appendix Figure S13. Quality control of AAV vector preparations. **page 17**

Appendix Tables

Appendix Table S1. Passive properties of hippocampal neurons transduced with blue light activated ChRs. **page 18**

Appendix Table S2. Channelrhodopsin variants. **page 19**

Appendix Table S3. List of primers used for ChR mutant generation. **page 20**

Appendix Figures

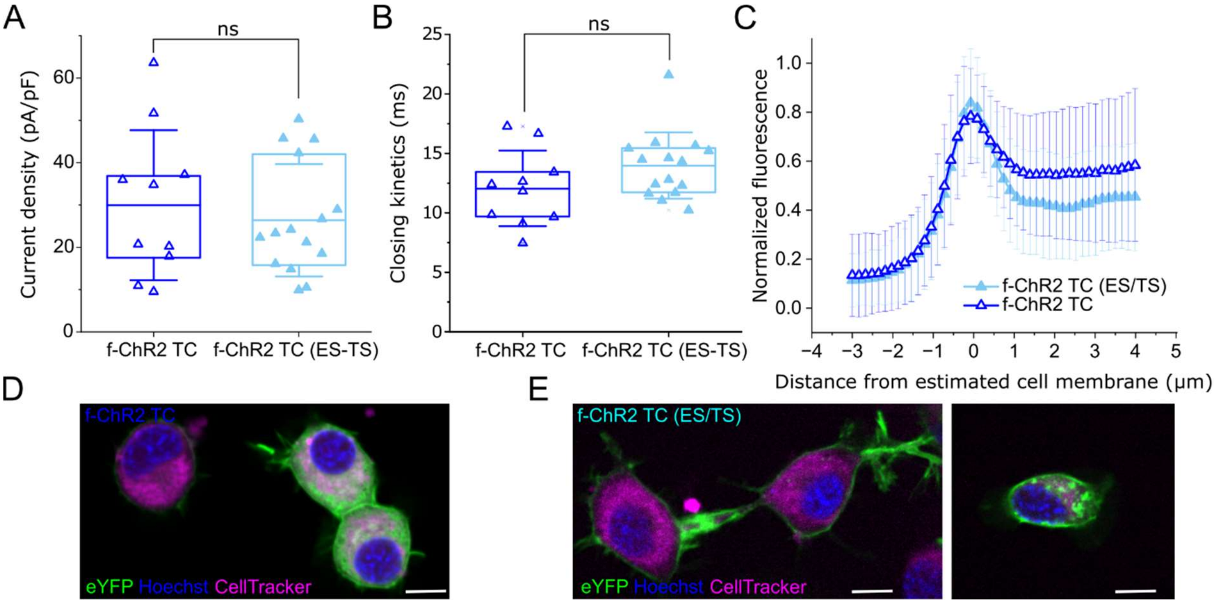

Appendix Figure S1. Effect of the C-terminal fusion of targeting motifs on the plasma membrane expression of f-ChR2 TC. A-B, Comparison of current densities (**A**) and closing kinetics (**B**) of f-ChR2 TC and f-ChR2 TC (ES/TS). The whole-cell patch-clamp measurements were performed at RT (~ 22 °C) in NG cells 2 to 3 days after transient transfection. τ_{off} values were determined by monoexponential fits to photocurrents evoked by 3 ms light pulses (λ = 473 nm, ~ 30 mW/mm^2) at a membrane potential of -60 mV. The statistical comparison was performed by unpaired t-tests (f-ChR2 TC: n = 10 vs f-ChR2 TC (ES/TS): n = 15; ns: p = 0.5719, p = 0.1231). **C,** Line profile analysis of single cells from three different transfections. Plots show means ± SDs for f-ChR2 TC: n = 91 and f-ChR2 TC (ES/TS): n = 51. **D-E**, Confocal images of exemplary transfected NG cells expressing f-ChR2 TC (**D,** panel D corresponds to Figure 1A) or f-ChR2 TC (ES/TS) (**E**) that are included in the quantification shown in **C**., EYFP fluorescence shown in green, nucleus stain (Hoechst, 1:2000) shown in blue, cytosol stain (CellTrackerTM, Invitrogen) shown in magenta. Single-plane images of the NG cells were collected by automated spinning disk confocal microscopy (CellVoyager CQ1, Yokogawa) at a magnification of 40 x. Scale bar 10 µm.

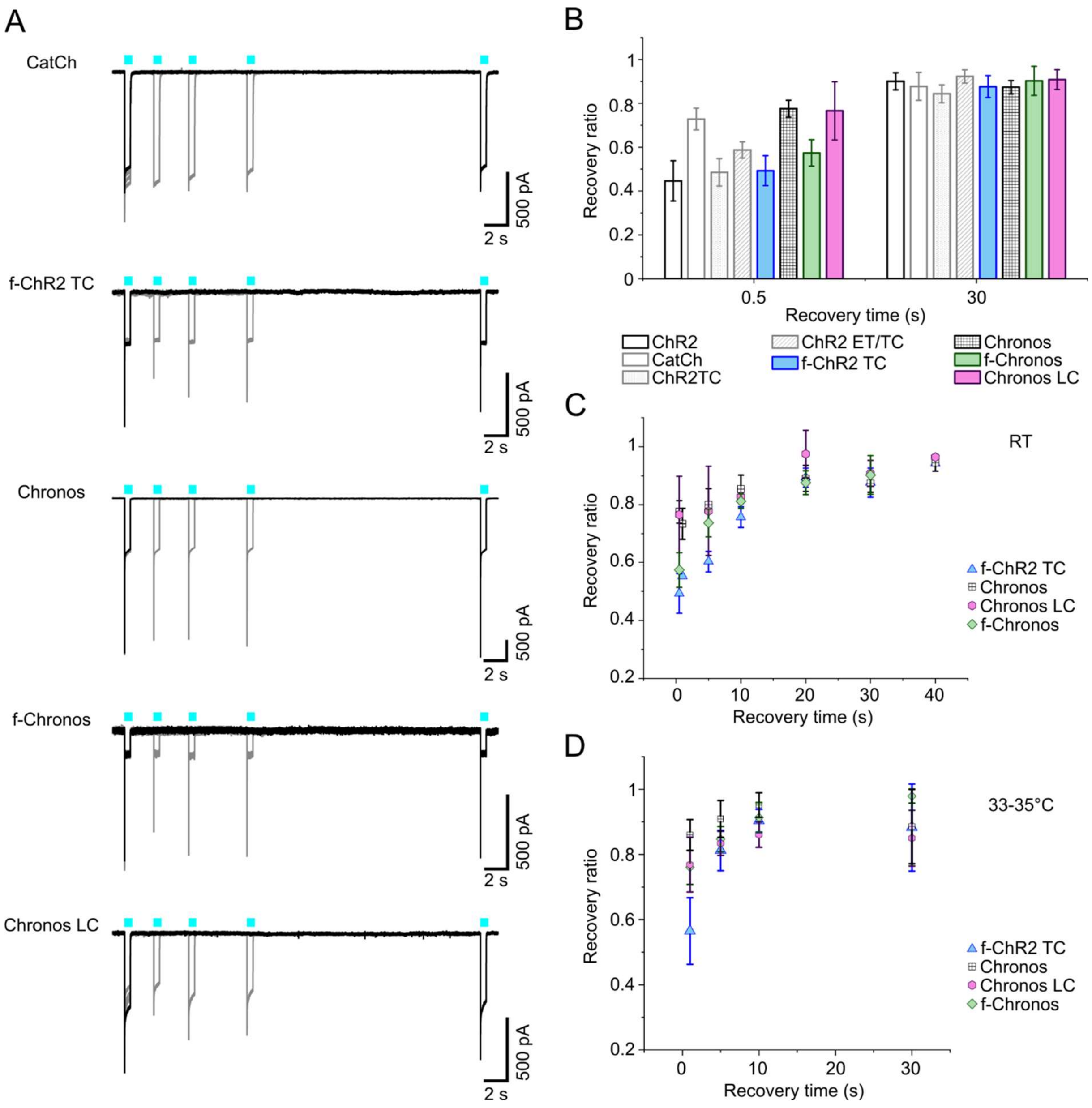

Appendix Figure S2. Recovery from photocurrent desensitization. A, Whole cell patch-clamp recordings from NG108-15 cells transiently expressing CatCh, f-ChR2 TC, Chronos, f-Chronos or Chronos LC. Overlaid representative traces of photocurrent peak recovery kinetics after applying two sequential 500 ms light pulses (488 nm; ~ 40 mW/mm^2) at different dark time intervals (1, 5, 10, and 30 sec). **B,** Peak photocurrent recovery ratios at the indicated time points. Bars display the mean ± SD. ChR2: n = 3; CatCh: n = 4; ChR2 TC: n = 4; ChR2 ET/TC: n = 4; f-ChR2 TC: n = 4 to 5; Chronos: n = 5; f-Chronos: n = 4; Chronos LC: n = 4. **C,** Peak photocurrent recovery ratios of selected variants were determined at different time intervals at RT. Dots display the mean ± SD. f-ChR2 TC: n = 3 to 5; Chronos: n = 2 to 5; f-Chronos: n = 2 to 4; Chronos LC: n = 3 to 4. **D,** Peak photocurrent recovery ratios of selected variants determined after indicated time intervals at a temperature of 33°C to 35 °C. Dots display the mean ± SD. f-ChR2 TC: n = 5 to 7; Chronos: n = 3 to 6; f-Chronos: n = 3; Chronos LC: n = 3.

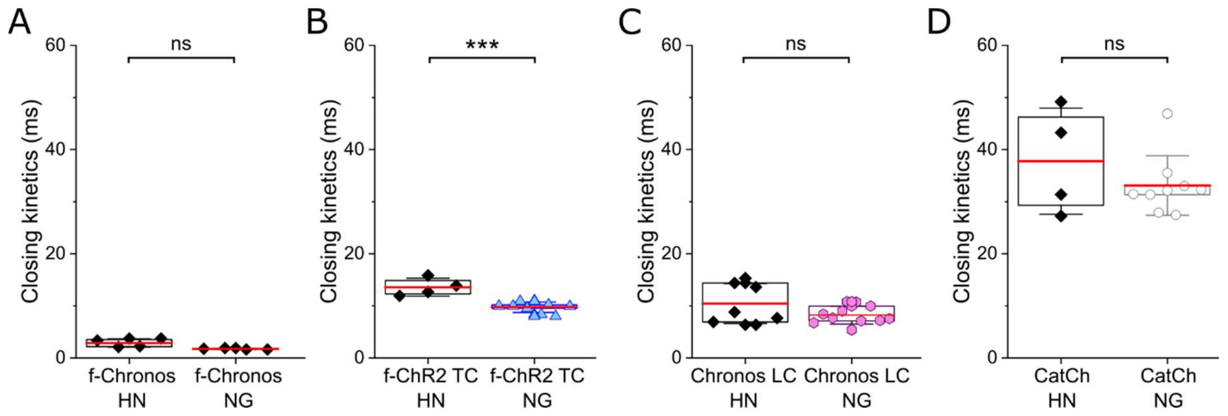

Appendix Figure S3. Comparison of ChR channel closing kinetics in rat hippocampal neurons (HN) and NG cells (NG). Shown is the statistical comparison of the channel closing kinetics of f-Chronos (**A**) by unpaired t-test with Welch's correction (p = 0.0656); f-ChR2 TC (**B**) by unpaired t-test (p = 0.0003); Chronos LC (**C**) by unpaired t-test with Welch's correction (p = 0.1930), and CatCh (**D**) by Mann-Whitney test (p = 0.8252). CatCh (HN): n = 4; CatCh (NG): n = 9; f-ChR2 TC (HN): n = 4; f-ChR2 TC (NG): n = 9; Chronos LC (HN): n = 7; Chronos LC (NG): n = 11; f-Chronos (HN): n = 4; f-Chronos (NG): n = 4. Data taken from Figure 1B (NG cells) and Figure 2 (hippocampal neurons).

A

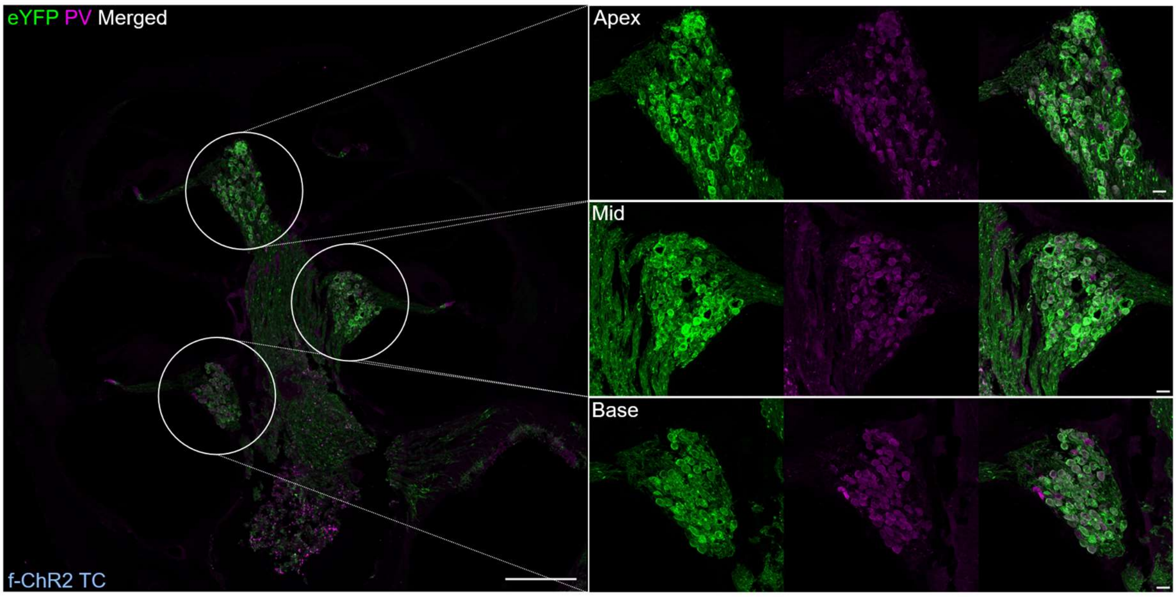

eYFP PV Merged
Apex
Mid
Base
f-ChR2 TC

B

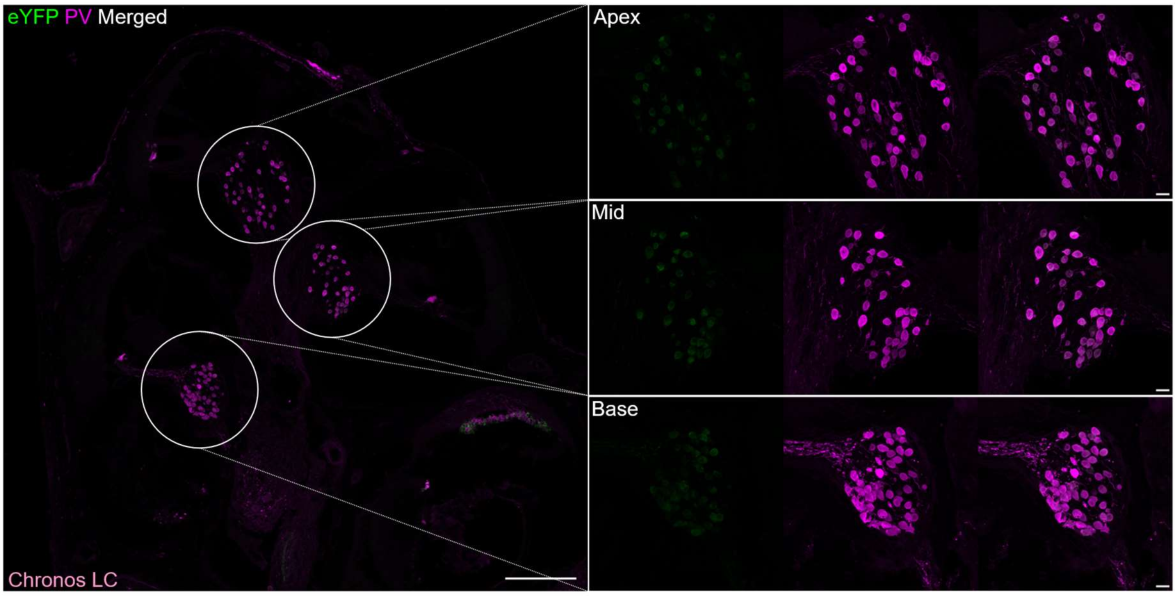

eYFP PV Merged
Apex
Mid
Base
Chronos LC

C

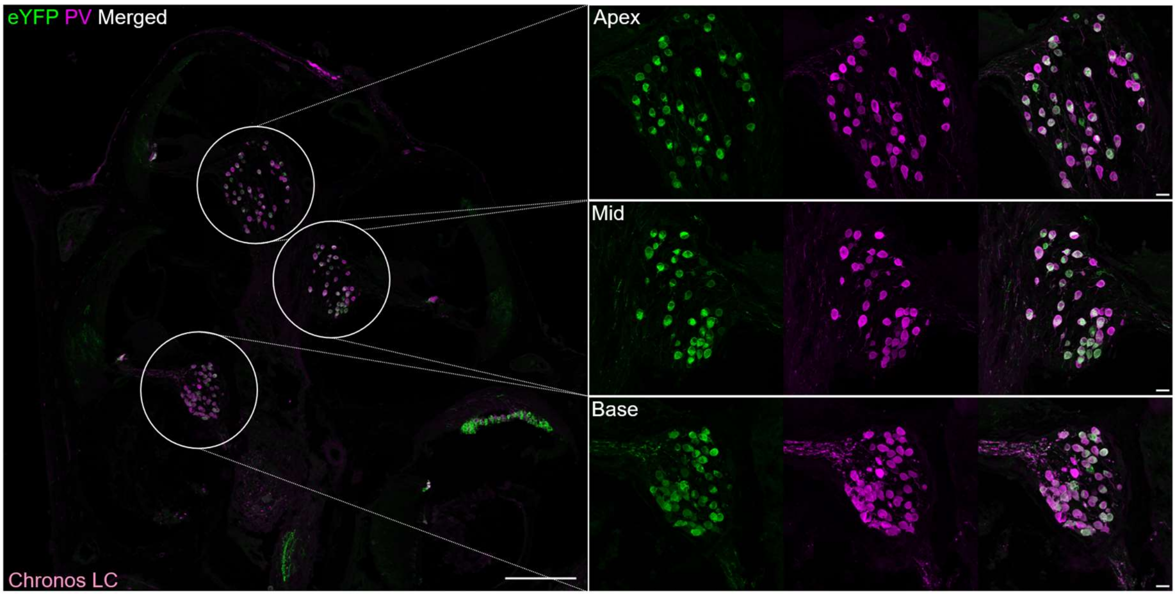

eYFP PV Merged
Apex
Mid
Base
Chronos LC

Appendix Figure S4. Immunohistochemical analysis of ChR expression in exemplary oABR-positive cochleae. A-C, 20x overview images of exemplary, mid-modiolar sliced cochleae, postnatally injected with AAV2/9_hSyn_ChR2(T159C/F219Y)-EYFP (**A**) and AAV2/9_hSyn_Chronos(L149C)-ES-EYFP-TS (**B-C**) shown on the left. 40 x magnifications of Apex, Mid, and Base shown on the right. Stainings: anti-parvalbumin (PV, SGN marker) and anti-GFP (ChR-EYFP). Scale bar at 20 x = 200µm and at 40 x = 20 µm.

A

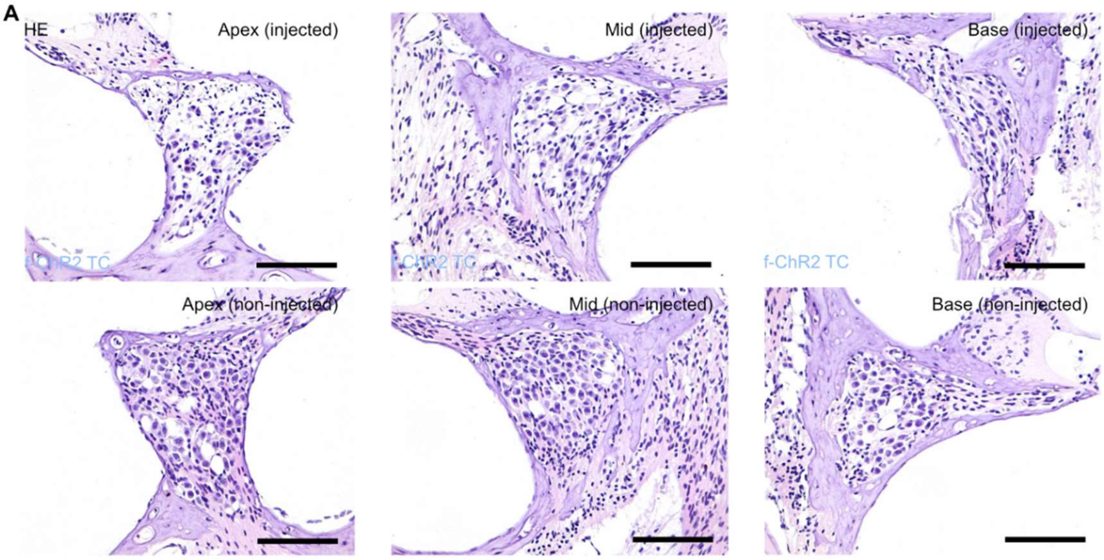

B

Spiral Ganglions			Turn	Chronos LC	Chronos LC	f-ChR TC	f-ChR TC	f-ChR TC
	neuron density	injected cochlea	a	1	1	3	2	1
			m	1	3	3	2	2
			b	2	3	3	3	2
		non-injected cochlea	a	3	4	3	3	3
			m	4	4	4	3	4
			b	4	4	4	4	4

Spiral Ganglions			Turn	Chronos LC	Chronos LC	f-ChR TC	f-ChR TC	f-ChR TC
	interstital vacuolation	injected cochlea	a	4	4	2	3	4
			m	4	2	2	3	3
			b	3	2	2	2	3
		non-injected cochlea	a	1	0	1	1	1
			m	0	0	0	1	0
			b	0	0	0	0	0

0	none
1	< 25 %
2	26-50 %
3	51-75 %
4	76-100 %

C **D**

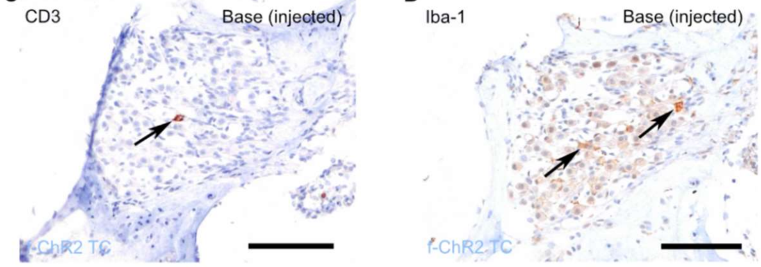

Appendix Figure S5. Histological and immunohistochemical analysis of cochlear paraffin-sections. A, Exemplary mid-modiolar HE-stained sections of apical, medial and basal turns of an animal injected with f-ChR2 TC. **B**, Quantification of neuronal density and interstitial vacuolation (optically empty matrix) for left = injected and right = non-injected (contralateral) cochleae of animals from the f-ChR2 TC (n = 3) and Chronos LC (n = 2) groups. **C,** Staining for CD3 lymphocytes of a left, basal turn of an animal from the f-ChR2 TC group. Arrow indicates a CD3 positive lymphocyte. **D,** Staining for Iba-1 (macrophages) of a left, basal turn of the same animal from the f-ChR2 TC group as in **C**. Arrows indicate cell-associated signal enhancement of Iba-1 expression (not further quantified due to strong diffuse background staining). Scale bars = 100 µm.

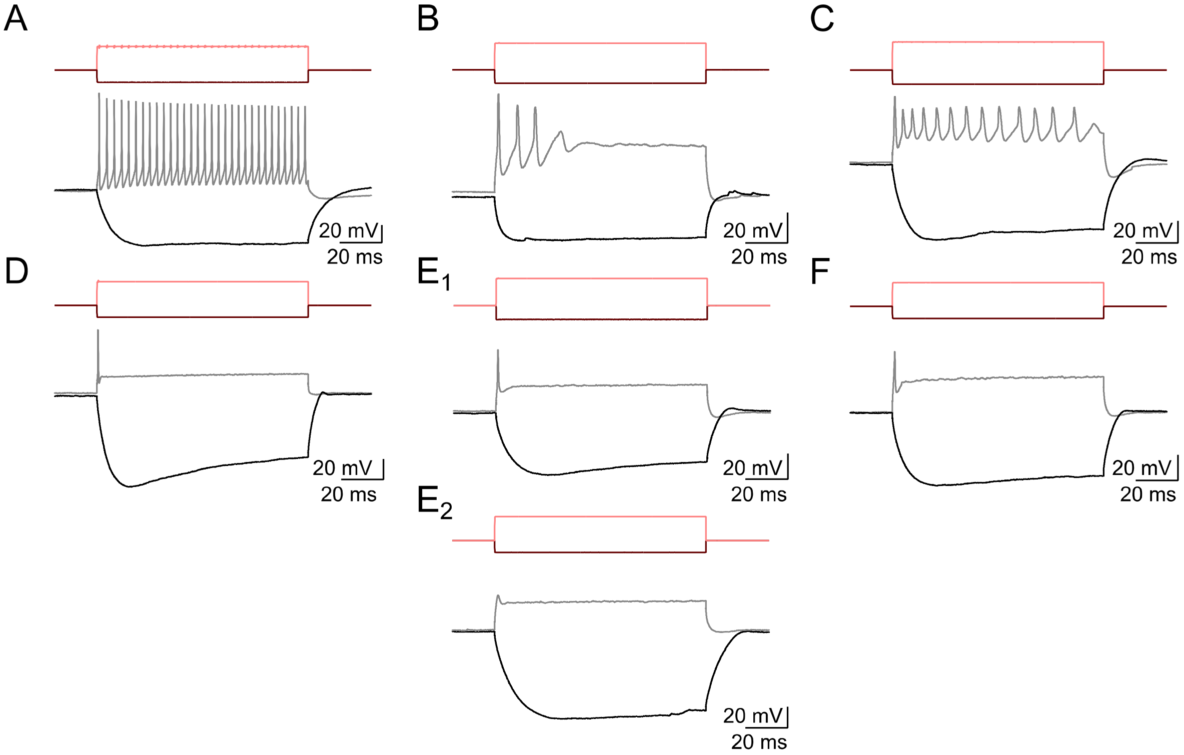

Appendix Figure S6. Characteristic firing patterns of AVCN neurons in response to 100 ms depolarizing current injection. Shown is tonic firing of a stellate cell (**A**) and phasic firing of a bushy cell (**D**) upon current injection. Measurements with an intracellular sodium channel blocker (1 mM QX-314) into the pipette solution immediately after establishing whole-cell configuration (**B-C:** stellate cells; **E-F:** bushy cells) and 30 min after establishing whole-cell configuration (**E_2**). Injection of hyperpolarizing currents results in the activation of hyperpolarization-activated (I_h) current (Cao et al. 2007; Rodrigues and Oertel 2006)

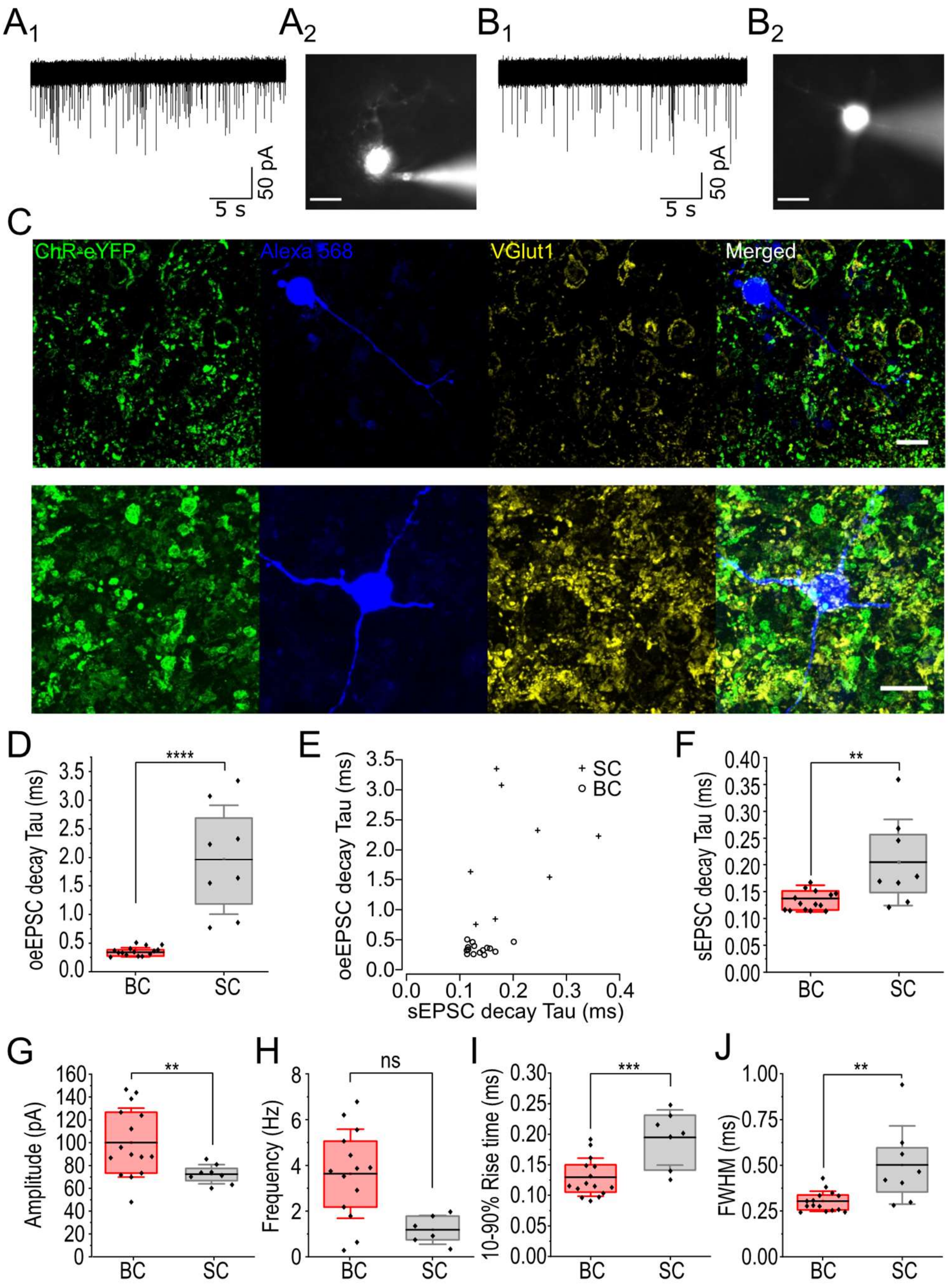

Appendix Figure S7. Classification of principal cells in the cochlear nucleus based on their spontaneous excitatory postsynaptic currents and cell morphology. A-B, Exemplary traces of spontaneous excitatory postsynaptic currents (sEPSCs) from a bushy cell (**A_1**) and from a stellate cell (**B_1**). Representative fluorescent image of a bushy cell (**A_2**) and a stellate cell (**B_2**) filled with Alexa 568. Bushy cells are characterized by short bushy-like dendrites branching next to the cell soma and are innervated by large endbulb of Held. Stellate cells have long dendrites (more than 2 usually) and receive bouton-like excitatory inputs from the auditory nerve terminals. **C**, Maximal z-projections of confocal image stacks of sagittal slices fixed and immunolabelled for EYFP (green) for f-ChR2 TC-EYFP localization, and context marker VGluT1 (yellow) as presynaptic vesicular transporter. Patched cells were filled with fixable Alexa 568 (blue). Scale bar = 20 µm. **D**, Spontaneous and optically evoked EPSCs decay kinetics show two clear populations that we classified as bushy cells (oeEPSC decay lower than 0.5 ms) and stellate cells (oeEPSC decay higher than 0.5 ms), as previously reported in (Isaacson and Walmsley 1995; Cao and Oertel 2010). **E-I**, sEPSCs of previously classified cells as BC or SC (**D**) were analyzed in terms of amplitude (**E**), frequency of events (**F**), decay time (**G**), full width half maxima time

(**H**), and rise time taken from 10 % to 90 % of the event (**I**). Significant differences were detected between the two groups by Mann-Whitney test: **** $p < 0.0001$; ** $p = 0.0087$; ** $p = 0.0087$; $p = 0.2381$ (ns); *** $p = 0.0005$; ** $p = 0.0022$. Outliers were detected by ROUT test and excluded. Values obtained are in line with previous reports (Gardner et al. 1999; Lu et al. 2007; Cao and Oertel 2010). BC: number of animals = 9; number of cells = 16. SC number of animals = 6; number of cells = 8.

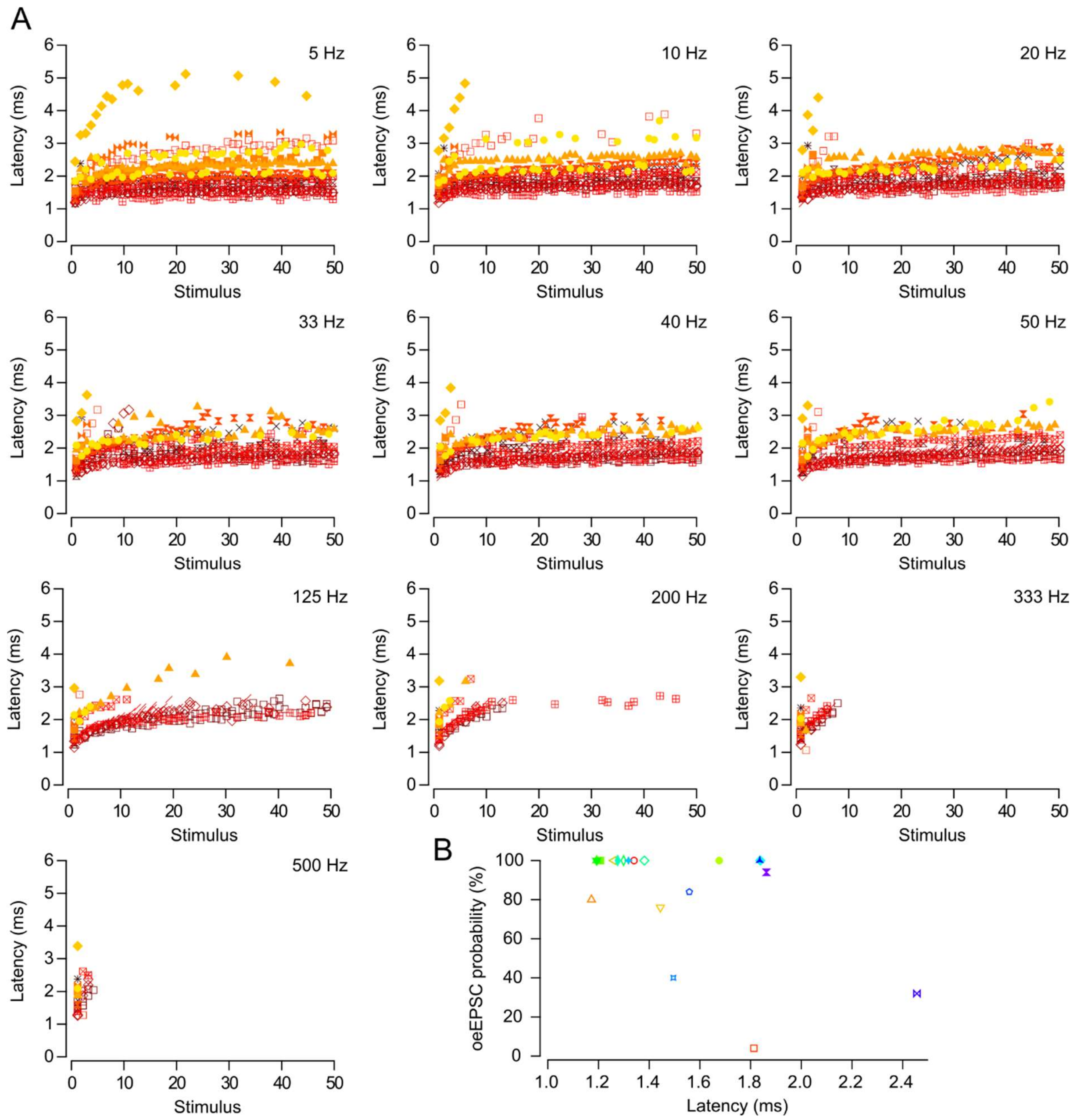

Appendix Figure S8. Spike latency increases over the train of light pulse stimulation. A, Quantification of latency by subtracting the time of stimulus onset from the time of the peak of the first oeEPSC (n = 15 BC; N = 9 slices from 9 mice). **B**, Cells with shorter latency show higher oeEPSC probabilities (frequency: 5 Hz, pulse length: 1 ms, λ = 488 nm, ~ 40 mW/mm^2). Note that low oeEPSC probability was not always correlating to long latencies, as shown exemplarily for the endbulb synapses depicted in red square when compared to other synapses with similar latency (displayed by cyan rhombus, purple hourglass icon (⧗) and blue triangular star).

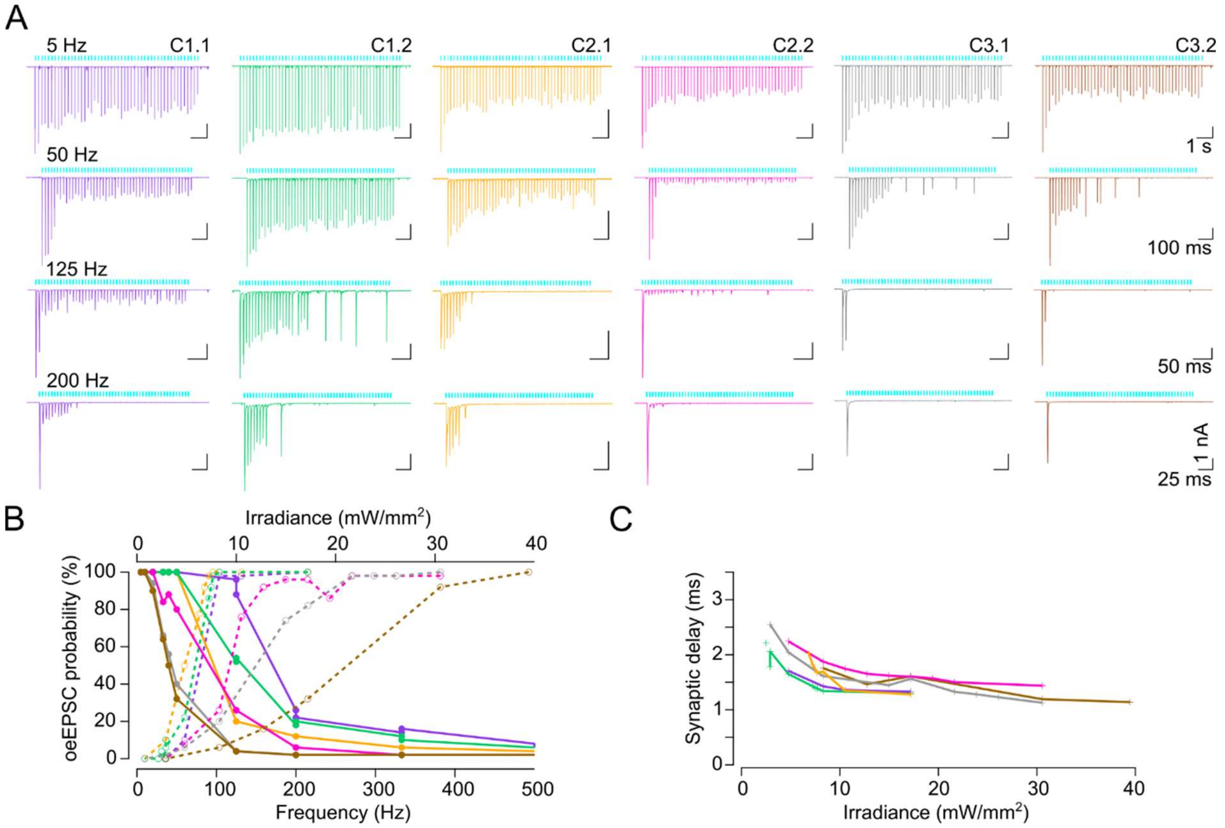

Appendix Figure S9. **Bushy cells with greater light sensitivity can follow higher frequencies of stimulation. A**, oeEPSCs in 6 different bushy cells, as indicated by the color code, receiving optogenetically evoked presynaptic inputs at different frequencies of light stimulation (pulse length: 1 ms, λ = 488 nm, ~ 40 mW/mm^2). Cells are displayed from left to right from higher to lower success rate at different frequencies. **B**, Dependence of oeEPSC probability on irradiance (dashed line and empty circles) at a frequency of 10 Hz and a pulse length of 1 ms and dependence of oeEPSC probability on frequency (continuous line and filled circles) (pulse length: 1 ms, λ = 488 nm, ~ 40 mW/mm^2). **C**, Quantification of synaptic delay dependent on frequency of stimulation (~ 40 mW/mm^2, 1 ms; continuous line). Cells plotted in **B**-**C** are the same.

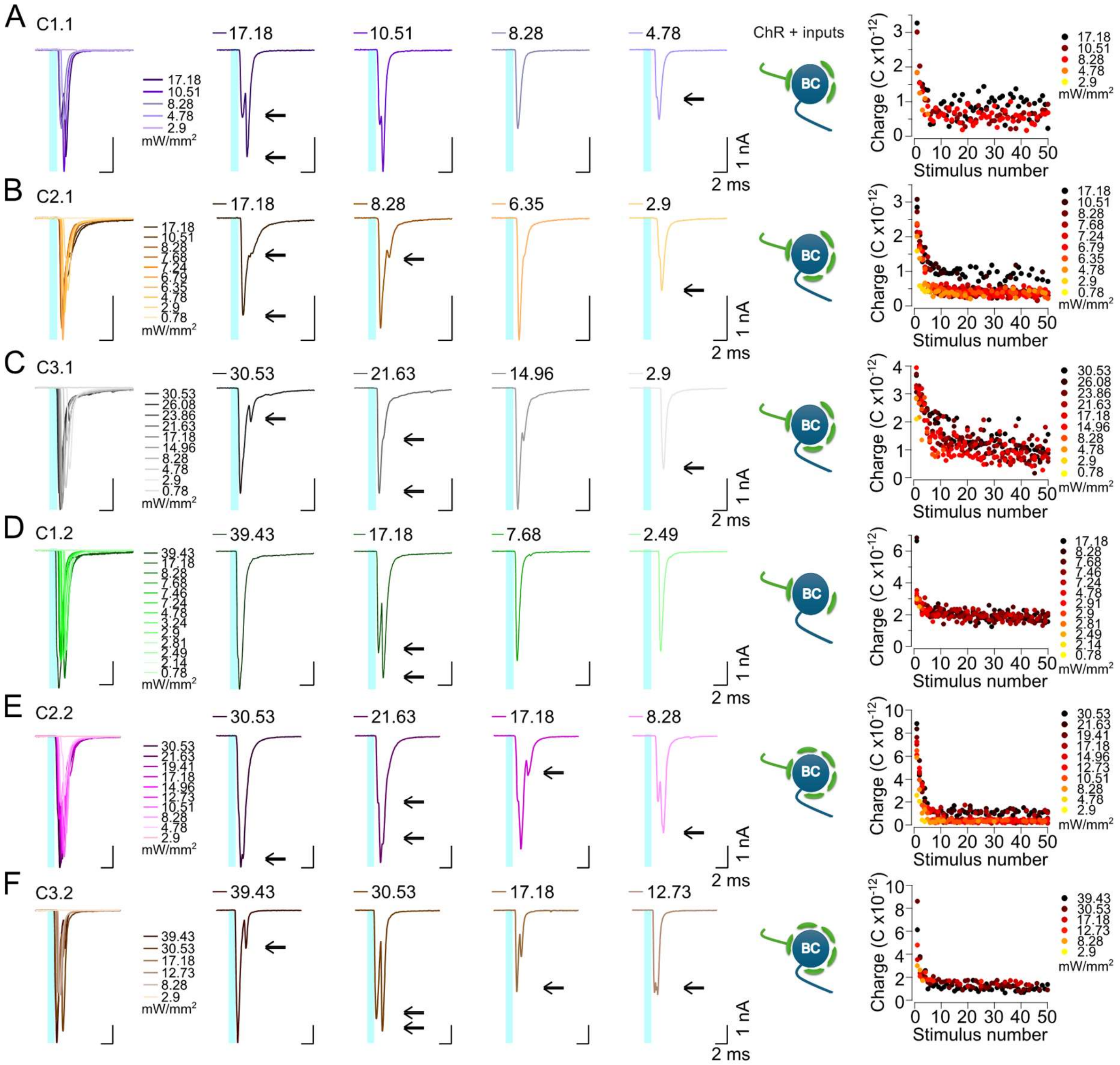

Appendix Figure S10. **oeEPSC measurements at different light intensities.** Shown are exemplary traces recorded in 6 different bushy cells (**A-F**). Overlapped oeEPSCs showing the dependence of amplitude and synaptic delay on light intensity (panels on the left). For clarity, selected traces are shown separately (panels in the middle). Dependence of oeEPSC charge on stimulus number at different light intensities (panels on the right). The charge levels likely correlated to the number of synaptic inputs. Data taken from measurements shown in Appendix Fig. S9.

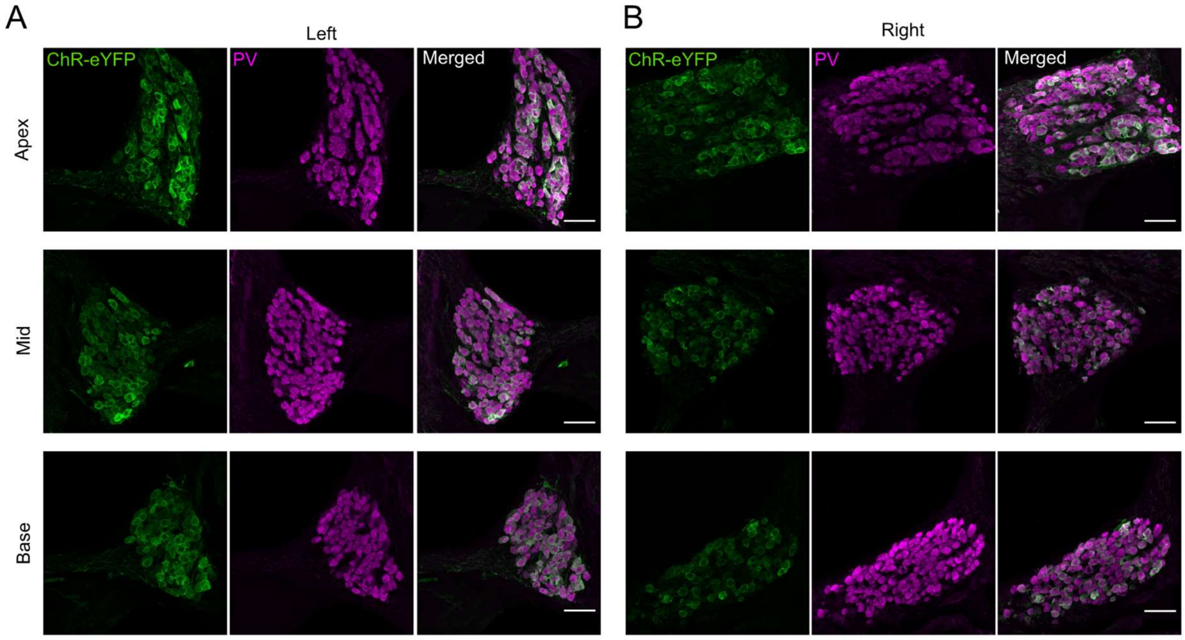

Appendix Figure S11. f-ChR2 TC expression in SGNs at P20 in postnatally injected mice. Projections of confocal mid-modiolar cryosections immunostainned for GFP (green) showing ChR-EYFP expression, and parvalbumin (magenta) as context marker in SGNs in three cochlear regions. Scale bar: 50 µm. Mice were injected unilaterally in the left side (**A**) but ChR expression was also detected in the right side (**B**) as previously reported in early postnatal injections (Bali et al. 2021).

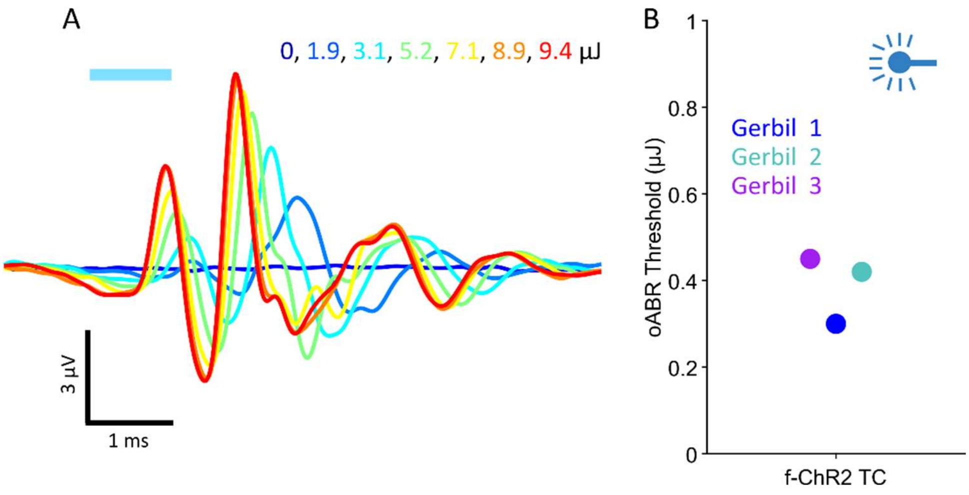

Appendix Figure S12. oABR shape and threshold for Mongolian gerbils expressing f-ChR2 TC in SGNs. **A**, Exemplary oABRs evoked by a laser fiber placed into the round window of a Mongolian gerbil, emitting blue light at 488 nm. 1 ms pulses were presented at 17 Hz. Different colors resemble radiant energy in µJ. **B**, Radiant energy thresholds for occurrence of an oABR for stimulus conditions in **A**. n = 3 gerbils (indicated by different colors). One gerbil did not show oABR responses. Icon in B was created in BioRender. Albrecht, N. (2025) https://BioRender.com/mz4pj4h.

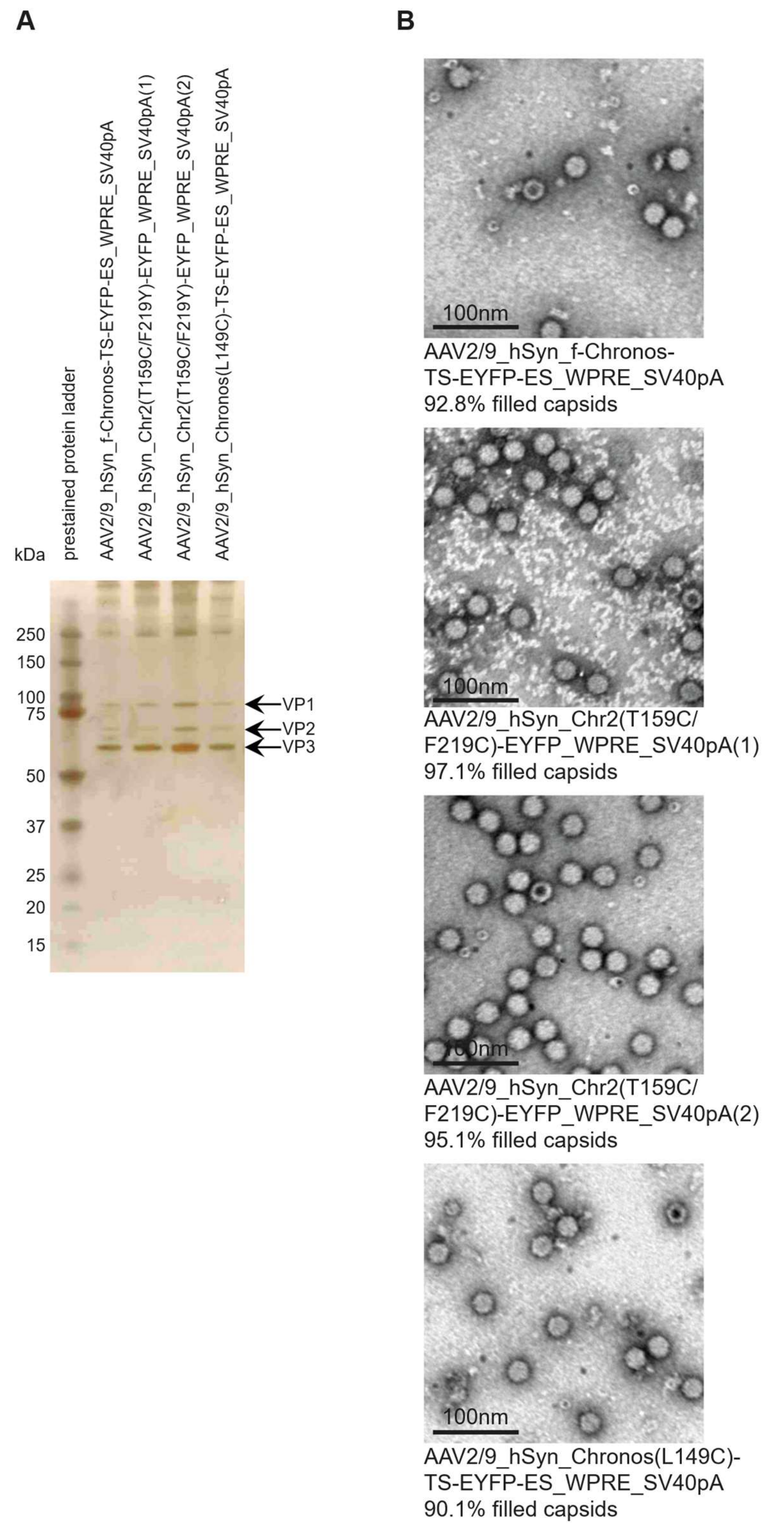

Appendix Figure S13. Quality control of AAV vector preparations. A, SDS-PAGE of AAV vector preparations used in this study depicted by silver impregnation. For virus protein (VP) size comparison Bio-Rad Dual Protein plus was used. **B**, Visualization of AAV particles using negative stainings in electron microscopy of AAV preparations used in this study. Here, AAV particles, 25 nm in size, filled with ChR-constructs depicted by white shapes can be distinguished from empty AAV particles containing a dark, negatively stained core. Additional smaller structures originate from serum protein or host cell contaminations. Percentages of AAV particle filling is calculated based on manual count in 10 random images per of AAV vector.

Appendix Tables

	Input resistance (GΩ)	Mean capacitance (pF)	Mean resting membrane potential (mV)
**f-Chronos (b)**	0.38 ± 0.27<br>(n = 15)	19.78 ± 9.59<br>(n = 15)	-62.87 ± 10.09<br>(n = 12)
**Chronos LC (d)**	0.22 ± 0.11<br>(n = 13)	23.99 ± 8.44<br>(n = 13)	-64.38 ± 8.09<br>(n = 13)
**f-ChR2 TC (g)**	0.35 ± 0.14<br>(n = 19)	21.38 ± 12.14<br>(n = 19)	-66.87 ± 9.94<br>(n = 18)
**CatCh (h)**	0.34 ± 0.26<br>(n = 15)	23.75 ± 9.75<br>(n = 15)	-64.4 ± 7.62<br>(n = 15)

Appendix Table S1. Passive properties of hippocampal neurons transduced with blue light activated ChRs. The passive properties of patched neurons after AAV transduction were comparable among groups.

	Species	Mutation(s)	Effect	Reference
**ChR2**	*Chlamydomonas reinhardtii*			(Nagel et al. 2003)
**CatCh**	*Chlamydomonas reinhardtii*	ChR2 L132C	Increased photocurrent amplitude. Reduced desensitization. Slower closing kinetics. Increased relative calcium permeability.	(Kleinlogel et al. 2011)
**ChR2 TC**	*Chlamydomonas reinhardtii*	ChR2 T159C	Increased photocurrent amplitude. Slower closing kinetics.	(Berndt et al. 2011)
**ChR2 ET/TC**	*Chlamydomonas reinhardtii*	ChR2 E123T/T159C	Increased photocurrent amplitude with fast closing kinetics.	(Berndt et al. 2011)
**f-ChR2**	*Chlamydomonas reinhardtii*	ChR2 F219Y	Faster closing kinetics.	(Mager et al. 2018)
**f-ChR2 TC**	*Chlamydomonas reinhardtii*	ChR2 T159C/F219Y	Increased photocurrent amplitude with fast closing kinetics.	This study
**Chronos**	*Stigeoclonium helveticum*			(Klapoetke et al. 2014)
**f-Chronos**	*Stigeoclonium helveticum*	Chronos F236Y	Ultra fast closing kinetics.	This study
**Chronos LC**	*Stigeoclonium helveticum*	Chronos L149C	Reduced desensitization. Slower closing kinetics.	This study
**f-Chronos LC**	*Stigeoclonium helveticum*	Chronos L149C/F236Y	Reduced desensitization. Slower closing kinetics.	This study
**Chrimson**	*Chlamydomonas noctigama*			(Klapoetke et al. 2014)
**f-Chrimson**	*Chlamydomonas noctigama*	Y261F/S267M	Faster closing kinetics.	(Mager et al. 2018)
**vf-Chrimson**	*Chlamydomonas noctigama*	K176R/Y261F/S267M	Faster closing kinetics.	(Mager et al. 2018)

Appendix Table S2. Channelrhodopsin variants. The table shows the ChR optimization strategy by the combination of point mutations with the shown effects.

construct	template	sequence of forward primer	sequence of reverse primer
Chronos F236Y	humanized Chronos	5’-TCATGGCCTACGTCTACTTCTGCTCTTGGC-3’	5’-GCCAAGAGCAGAAGTAGACGTAGGCCATGA-3’
Chronos L149C	humanized Chronos	5´-ACTTGCCCCGTGATCTGCATCCACCTGAGCAAC-3´	5´-GTTGCTCAGGTGGATGCAGATCACGGGGCAAGT-3´
Chronos L149C/F236Y	humanized Chronos F236Y	5´-ACTTGCCCCGTGATCTGCATCCACCTGAGCAAC-3´	5´-GTTGCTCAGGTGGATGCAGATCACGGGGCAAGT-3´
ChR2 T159C	humanized ChR2	5´-TCCGACAT CGGCTGTATCGTAT GGGG-3´	5´- CCCCAT ACGATACAGCCG ATGTCGGA-3´
ChR2 E123T/T159C	humanized ChR2 T159C	5´-CTGCGGTATGCCACTTGGCTGCTGAC-3´	5´-GTCAGCAGCCAAGTGGCATACCGCAG-3´
ChR2 F219Y/T159C	humanized ChR2 T159C	5´-TGGCTTGGCTGTACTTCGTGTCCTG-3´	5´-CAGGACACGAAGTACAGCCAAGCCA-3´

Appendix Table S3. List of primers used for ChR mutant generation.
