# Supplementary figures and images for: Channelrhodopsin variants for high-rate optogenetic neurostimulation at low light intensities
Source: EMBO Mol Med. 2025 Dec 9;18(2):462–91. doi: 10.1038/s44321-025-00350-z (PMC12905302; doi:10.1038/s44321-025-00350-z)

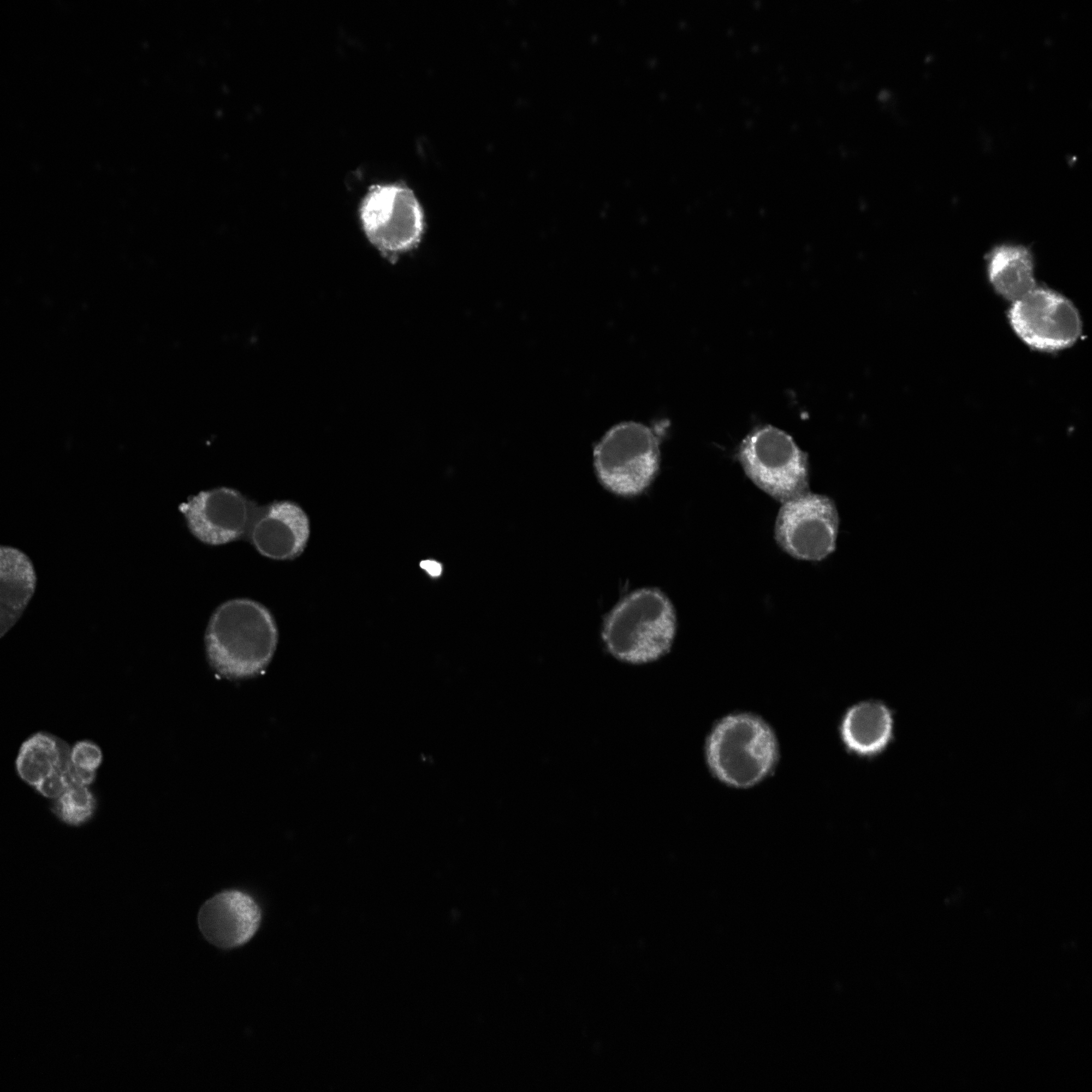

Supplement: Supplementary file 6 — Source data Fig. 1 [file 44321_2025_350_MOESM6_ESM.zip › Figure 1/1A/Fig1A_exemplary NG cells fChR2TC.tif]

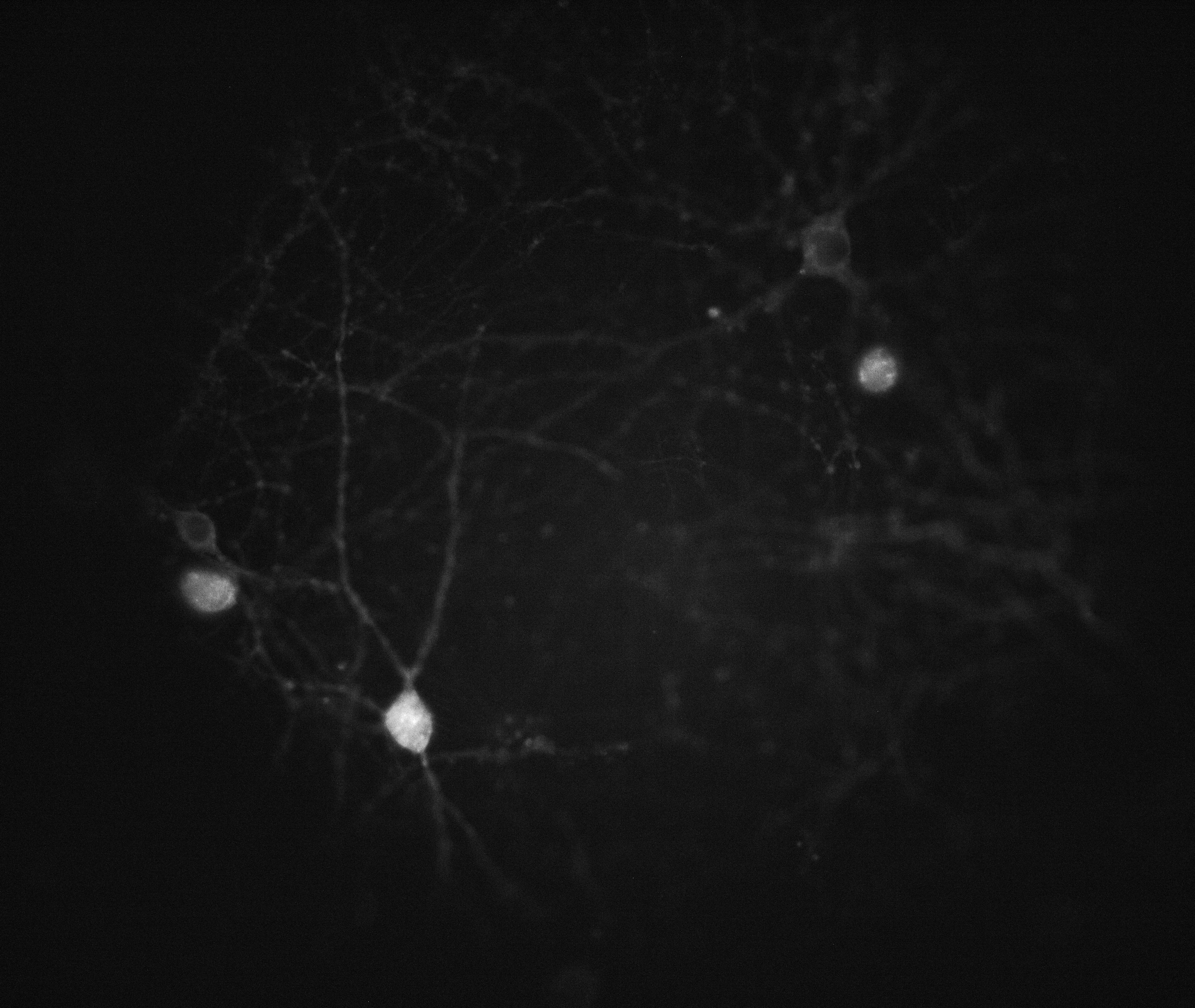

Supplement: Supplementary file 7 — Source data Fig. 2 [file 44321_2025_350_MOESM7_ESM.zip › Figure 2/2A/Fig2A_exemplary hippocampal neuron fChronos.tif]

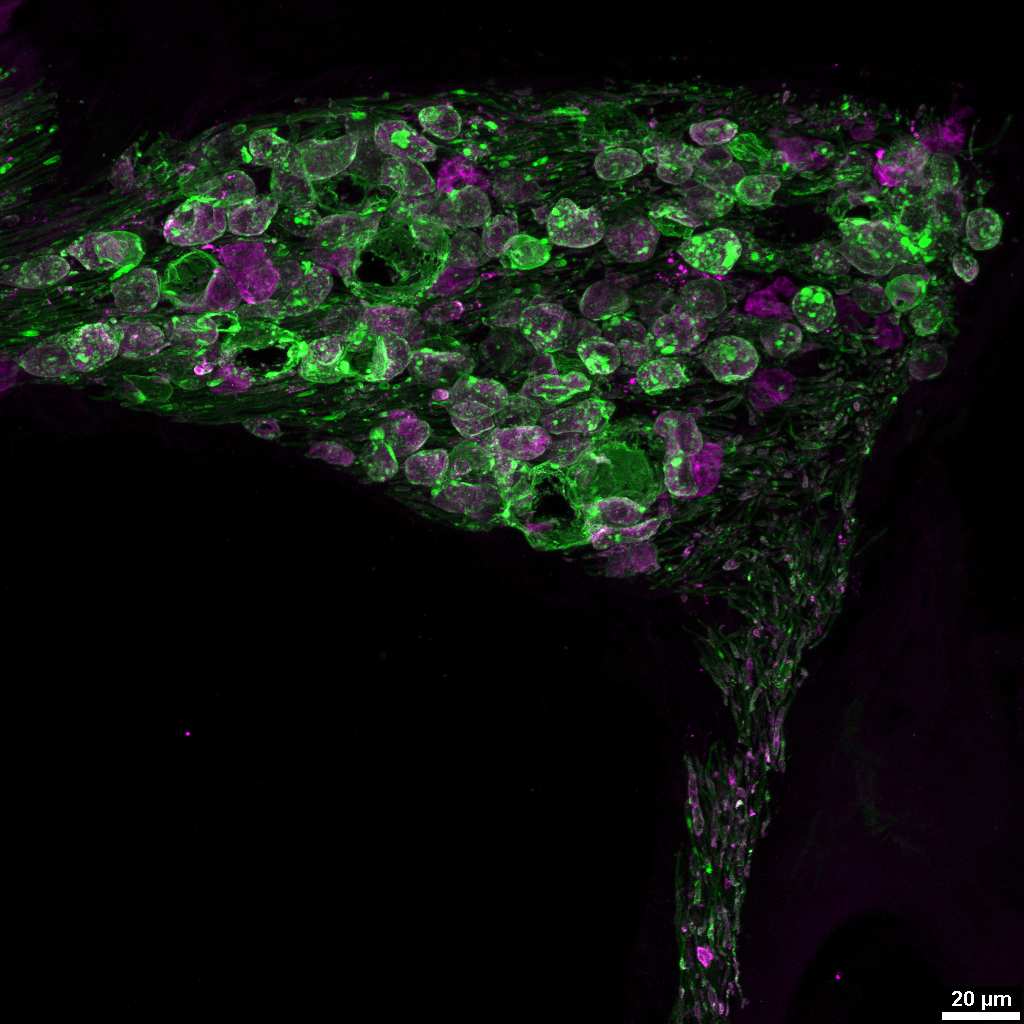

Supplement: Supplementary file 9 — Source data Fig. 4 [file 44321_2025_350_MOESM9_ESM.zip › Figure 4/Fig4A/f-Chr2 TC_apical turn_GFP-PV.tif]

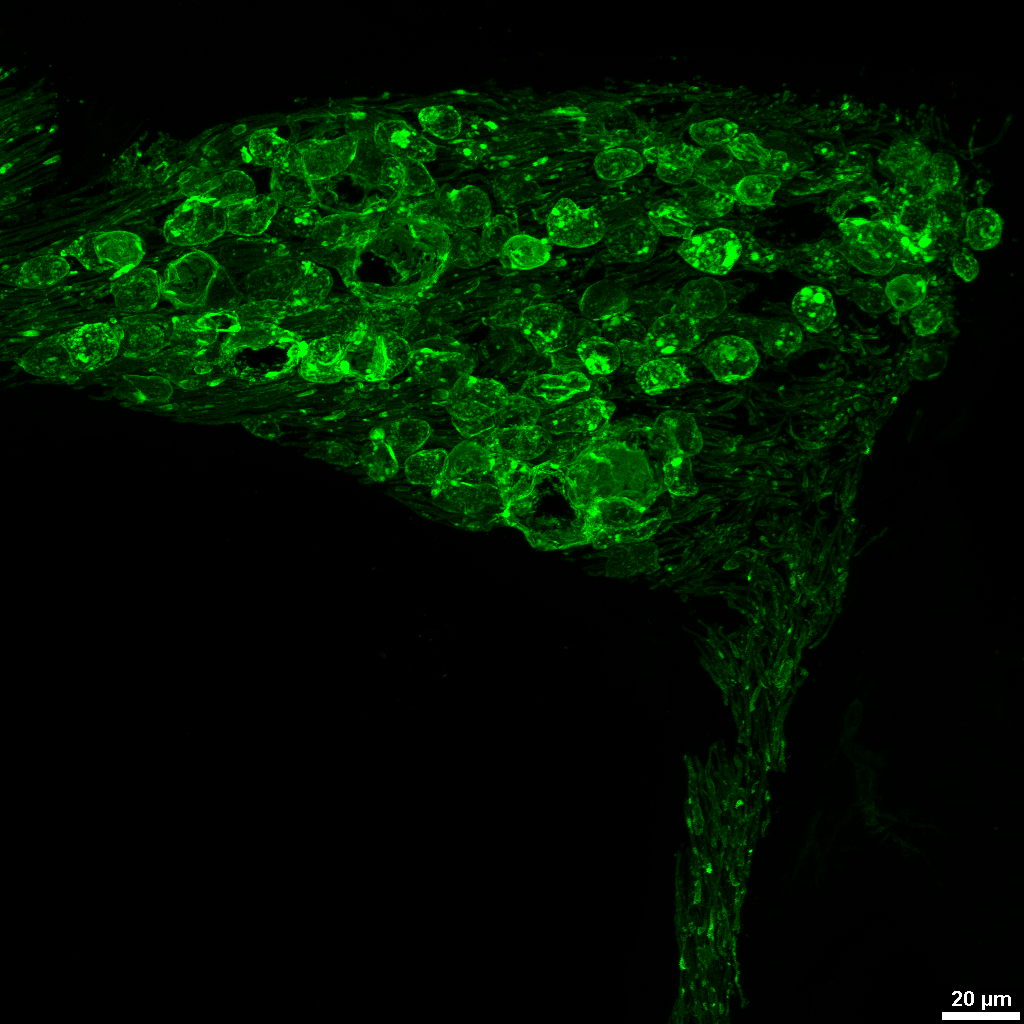

Supplement: Supplementary file 9 — Source data Fig. 4 [file 44321_2025_350_MOESM9_ESM.zip › Figure 4/Fig4A/f-Chr2 TC_apical turn_GFP.tif]

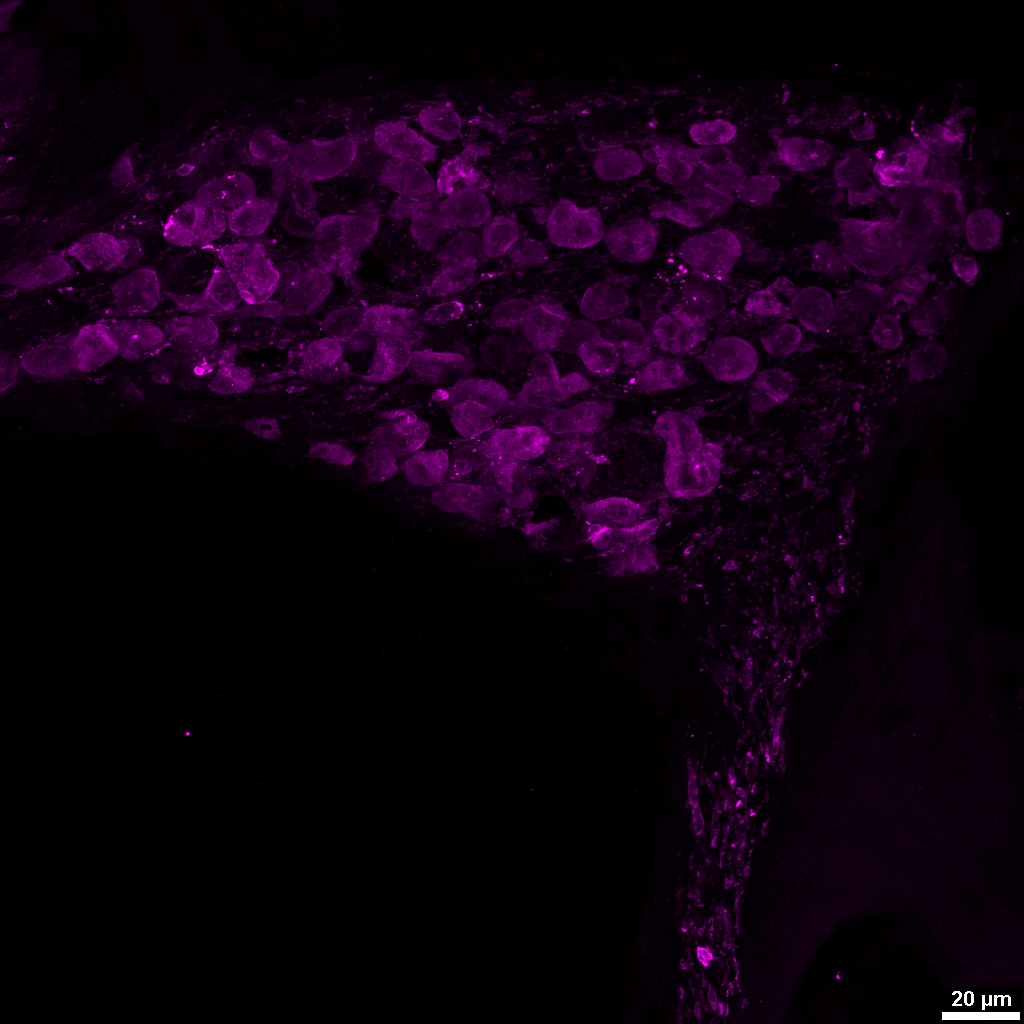

Supplement: Supplementary file 9 — Source data Fig. 4 [file 44321_2025_350_MOESM9_ESM.zip › Figure 4/Fig4A/f-Chr2 TC_apical turn_PV.tif]

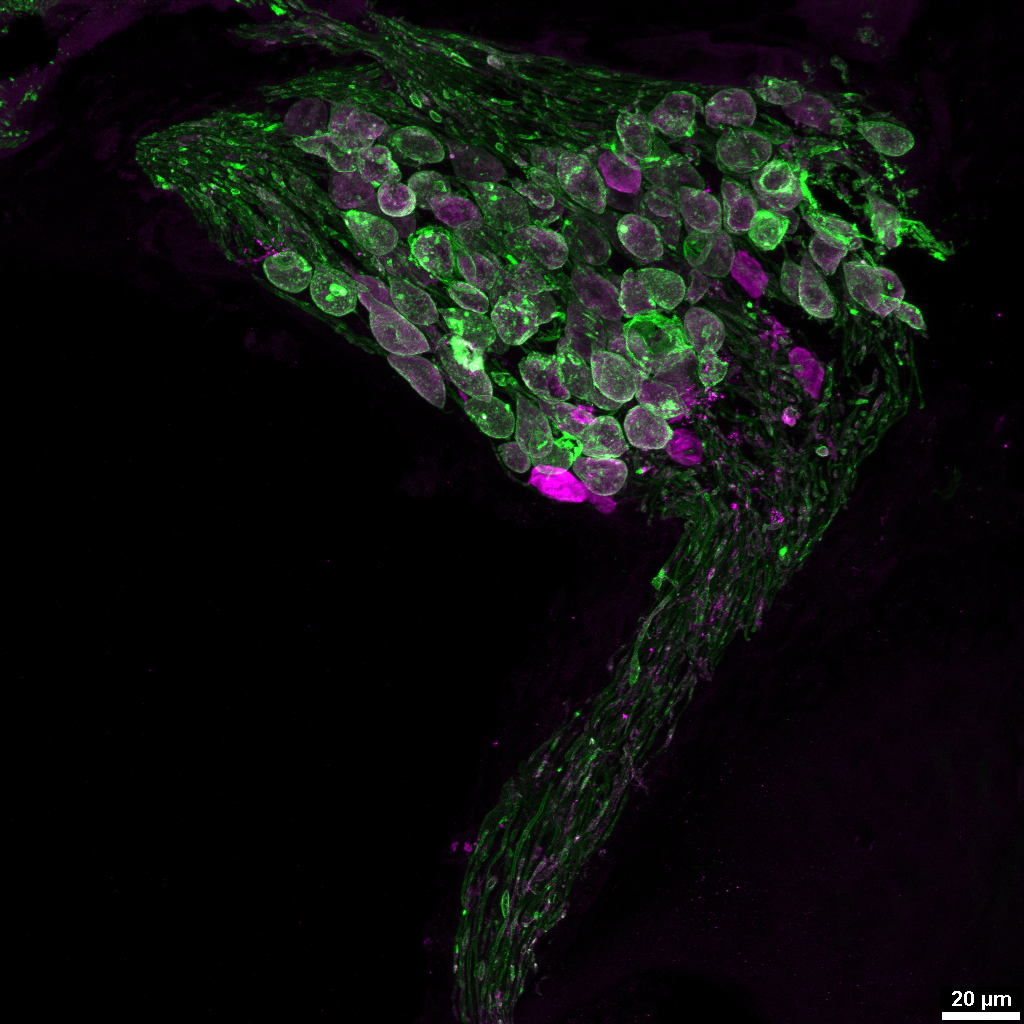

Supplement: Supplementary file 9 — Source data Fig. 4 [file 44321_2025_350_MOESM9_ESM.zip › Figure 4/Fig4A/f-Chr2 TC_basal turn_GFP-PV.tif]

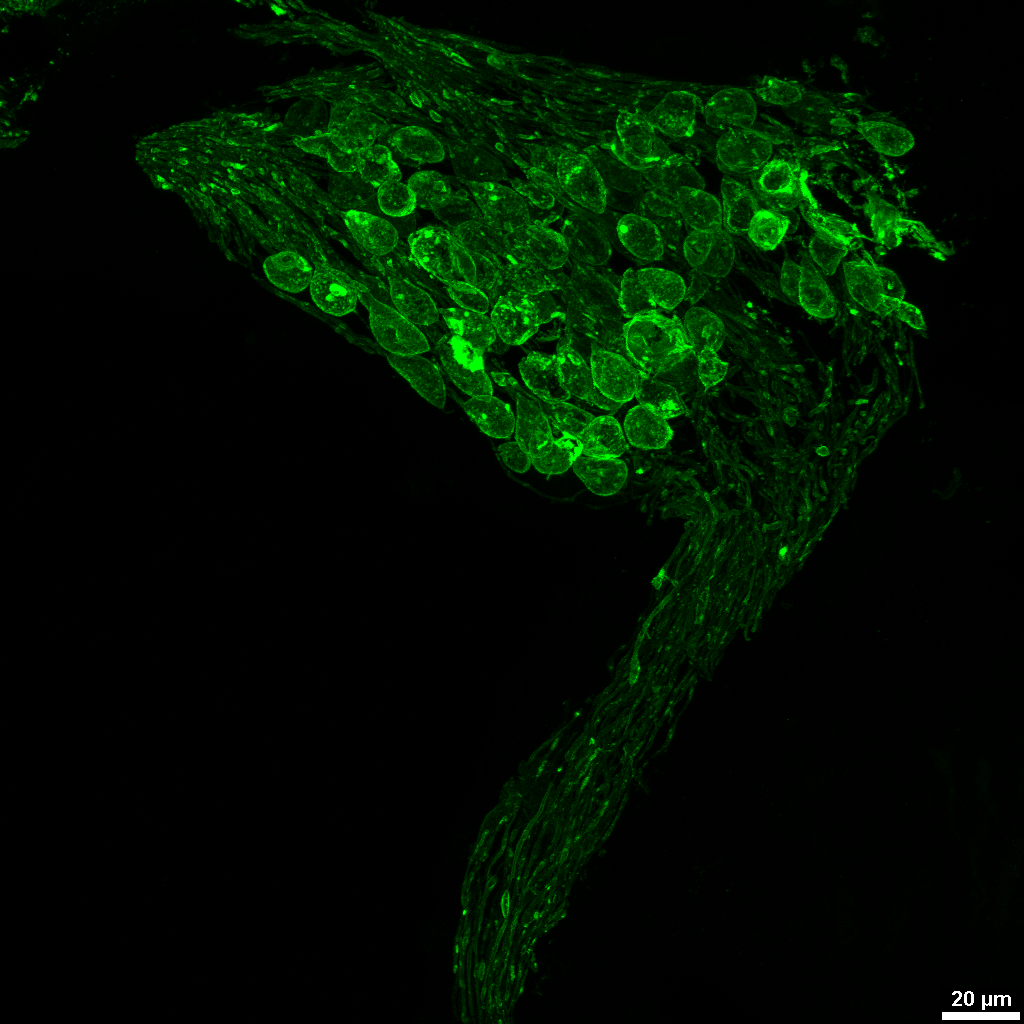

Supplement: Supplementary file 9 — Source data Fig. 4 [file 44321_2025_350_MOESM9_ESM.zip › Figure 4/Fig4A/f-Chr2 TC_basal turn_GFP.tif]

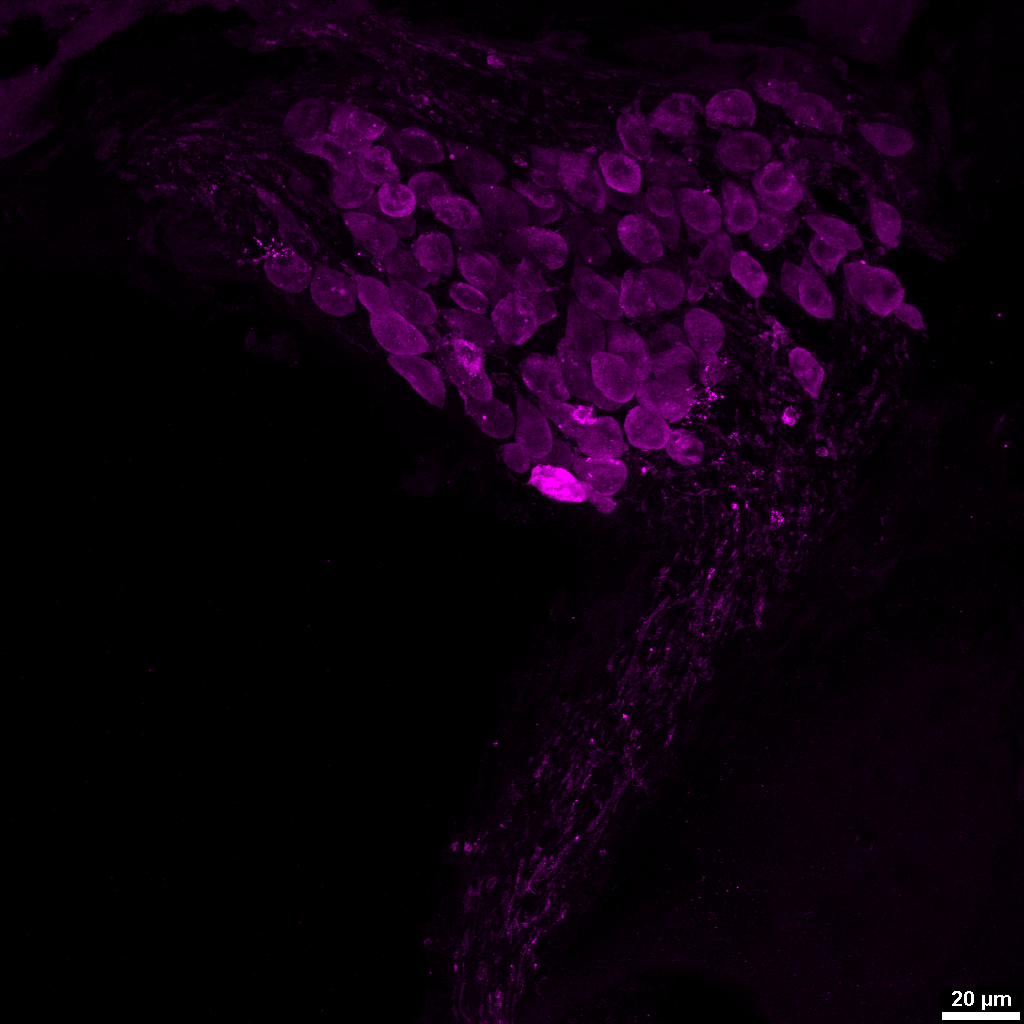

Supplement: Supplementary file 9 — Source data Fig. 4 [file 44321_2025_350_MOESM9_ESM.zip › Figure 4/Fig4A/f-Chr2 TC_basal turn_PV.tif]

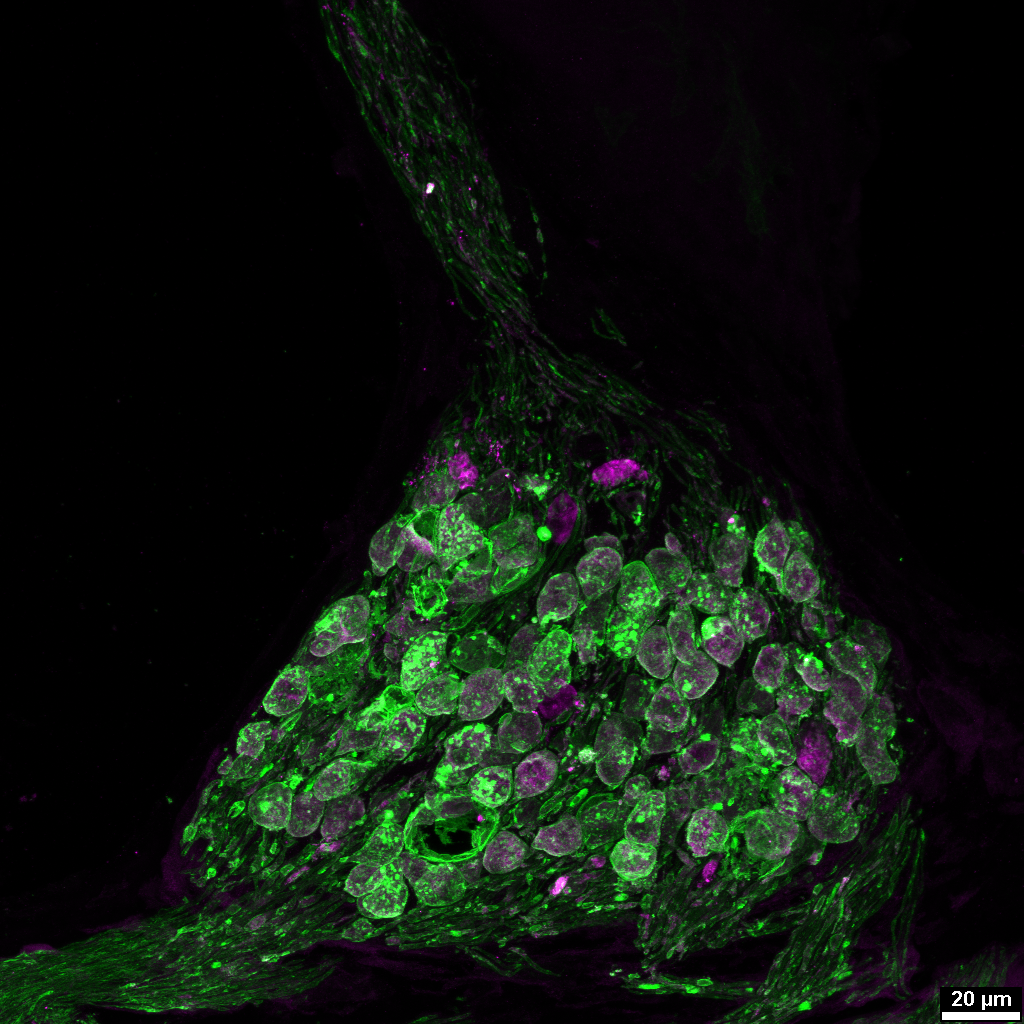

Supplement: Supplementary file 9 — Source data Fig. 4 [file 44321_2025_350_MOESM9_ESM.zip › Figure 4/Fig4A/f-Chr2 TC_medial turn_GFP-PV.tif]

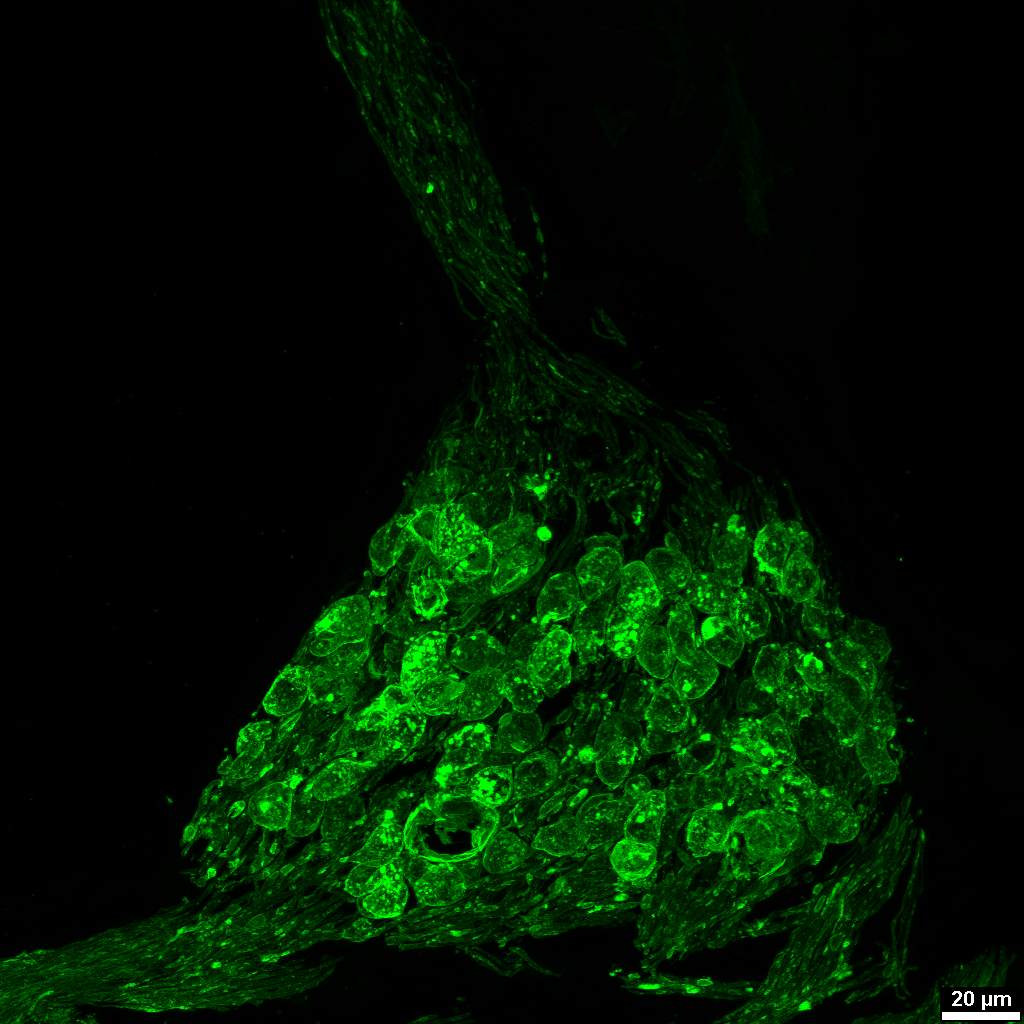

Supplement: Supplementary file 9 — Source data Fig. 4 [file 44321_2025_350_MOESM9_ESM.zip › Figure 4/Fig4A/f-Chr2 TC_medial turn_GFP.tif]

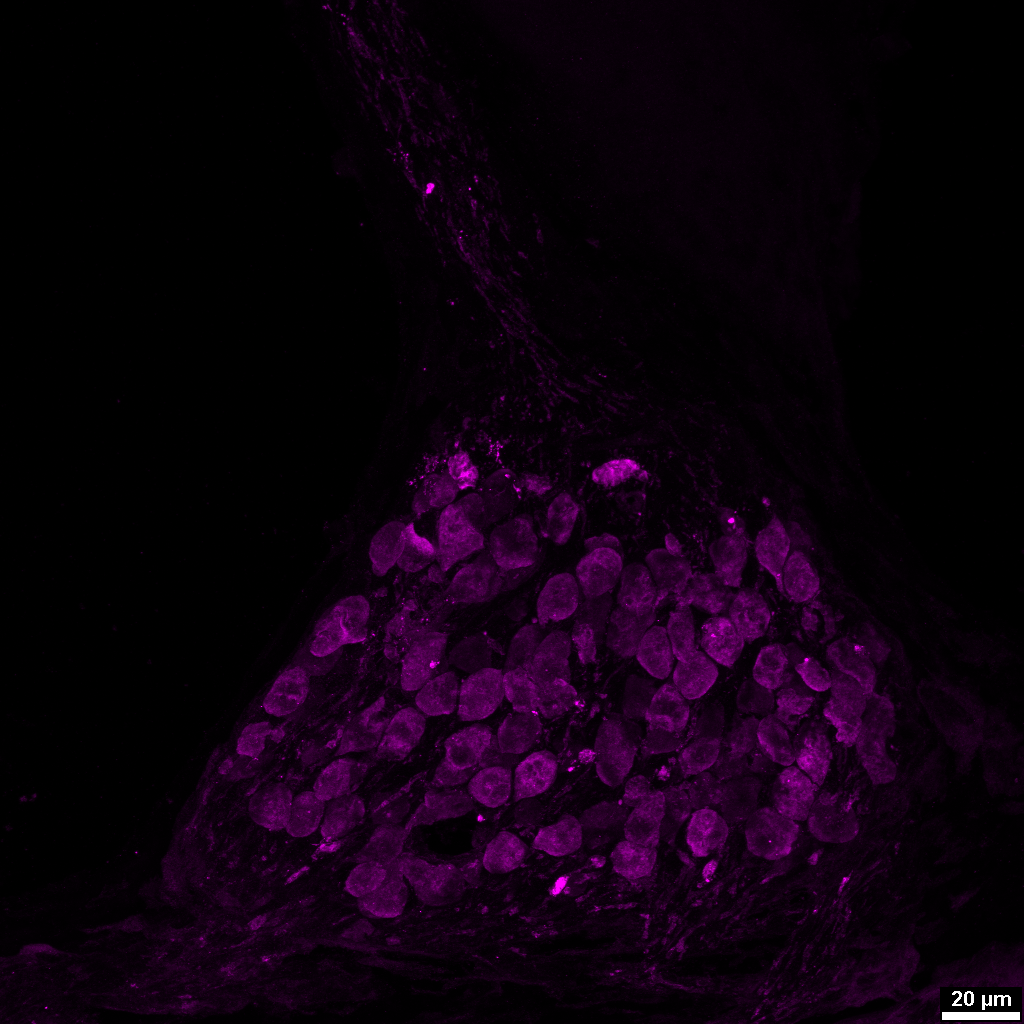

Supplement: Supplementary file 9 — Source data Fig. 4 [file 44321_2025_350_MOESM9_ESM.zip › Figure 4/Fig4A/f-Chr2 TC_medial turn_PV.tif]

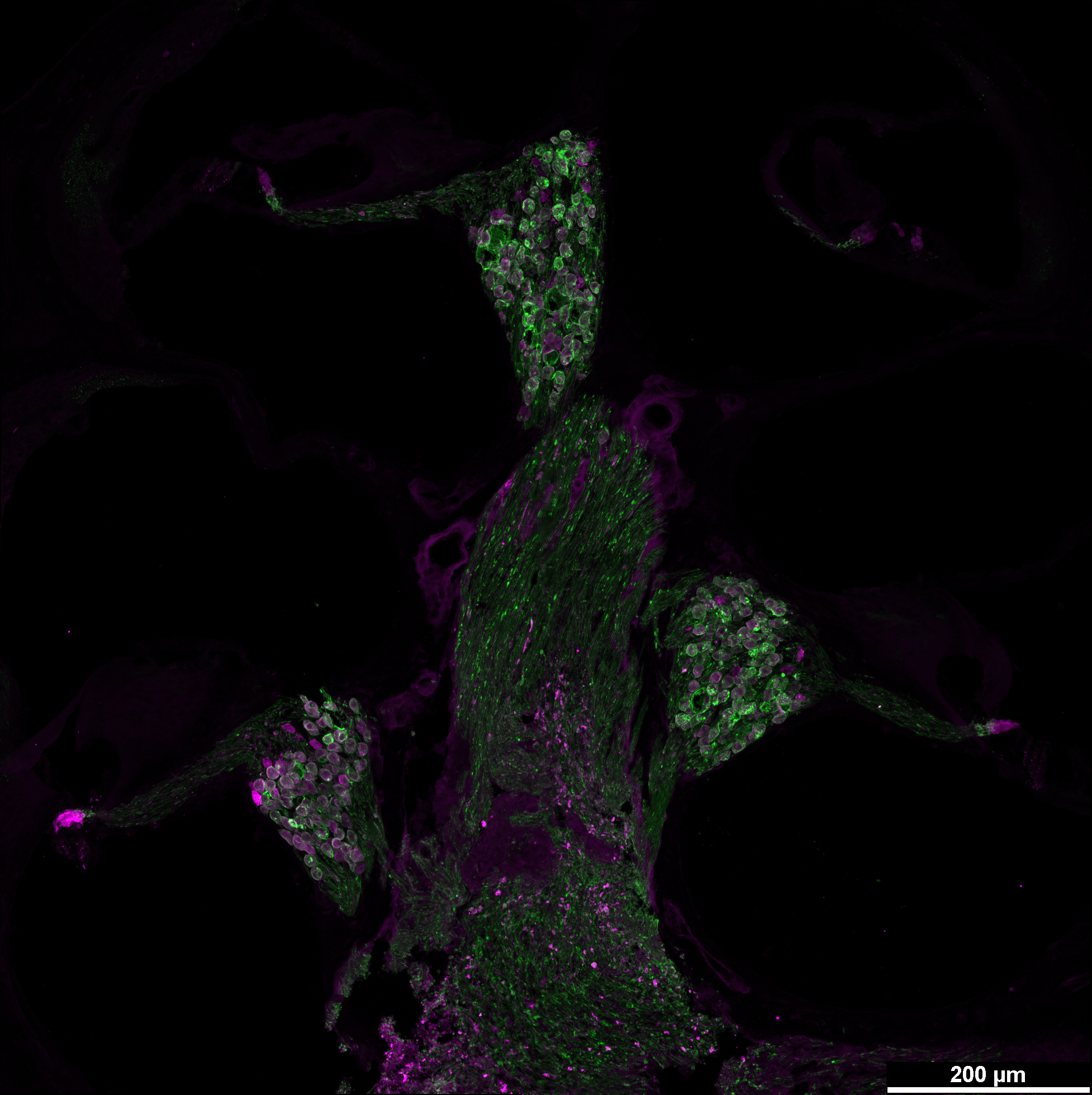

Supplement: Supplementary file 9 — Source data Fig. 4 [file 44321_2025_350_MOESM9_ESM.zip › Figure 4/Fig4A/f-Chr2 TC_overview_GFP-PV.tif]

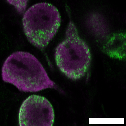

Supplement: Supplementary file 9 — Source data Fig. 4 [file 44321_2025_350_MOESM9_ESM.zip › Figure 4/Fig4E/Chronos LC_GFP-PV.tif]

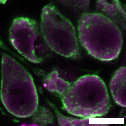

Supplement: Supplementary file 9 — Source data Fig. 4 [file 44321_2025_350_MOESM9_ESM.zip › Figure 4/Fig4E/f-ChR2 TC_GFP-PV.tif]

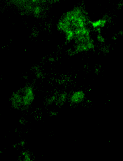

Supplement: Supplementary file 10 — Source data Fig. 5 [file 44321_2025_350_MOESM10_ESM.zip › Figure 5/Figure5B/Figure5B2.tif]

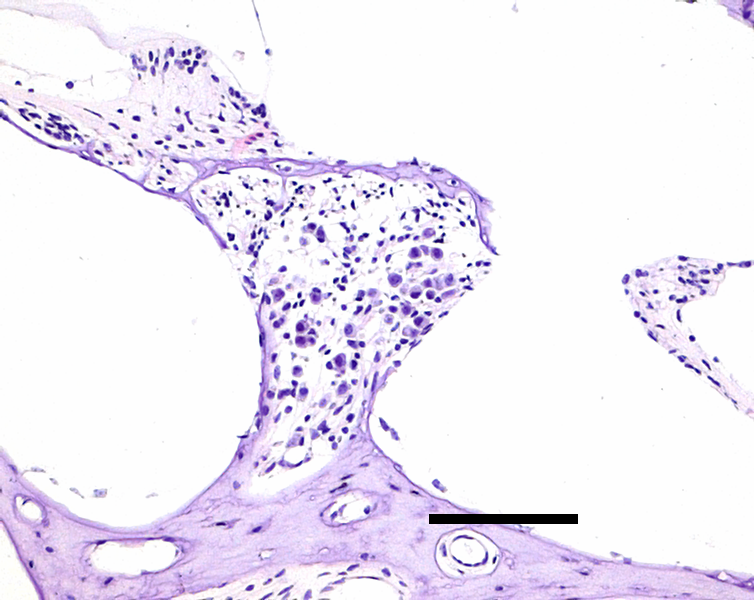

Supplement: Supplementary file 12 — Source Data Appendix Figure S5 [file 44321_2025_350_MOESM12_ESM.zip › Source_Data_Appendix_Figure_S5/Appendix_Figure_S5A_source-image-1-HE.tif]

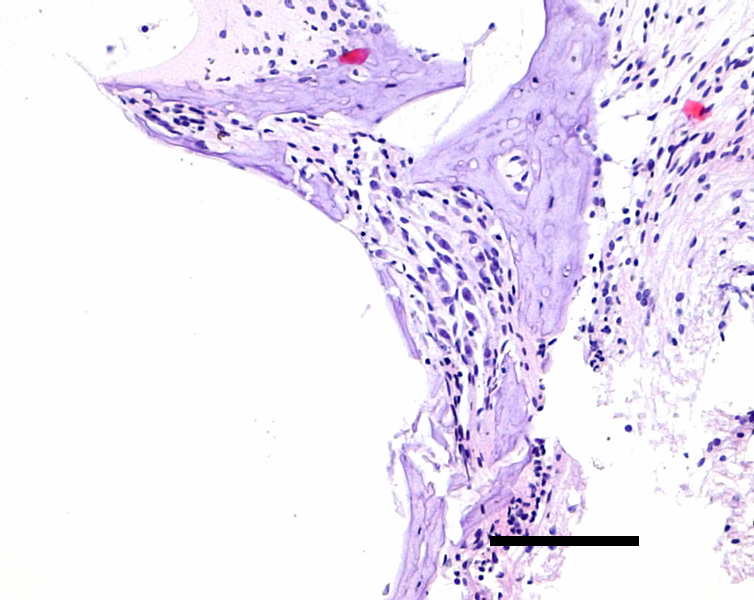

Supplement: Supplementary file 12 — Source Data Appendix Figure S5 [file 44321_2025_350_MOESM12_ESM.zip › Source_Data_Appendix_Figure_S5/Appendix_Figure_S5A_source-image-2-HE.tif]

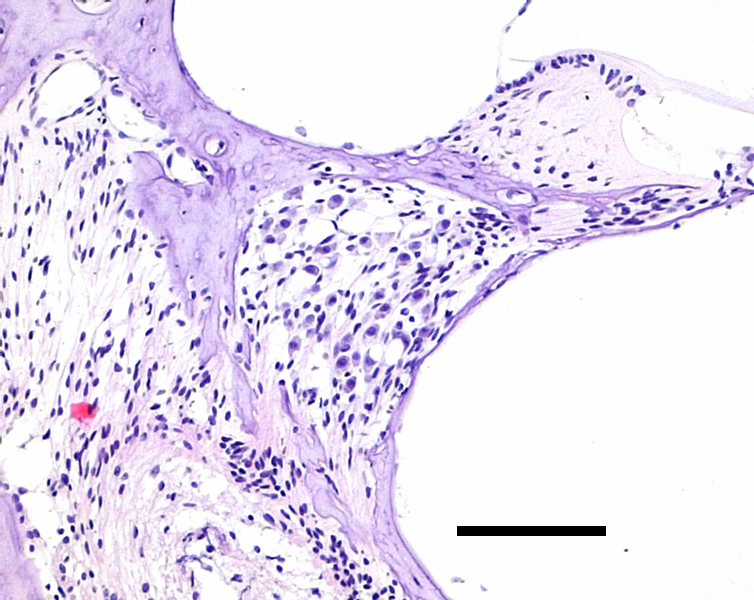

Supplement: Supplementary file 12 — Source Data Appendix Figure S5 [file 44321_2025_350_MOESM12_ESM.zip › Source_Data_Appendix_Figure_S5/Appendix_Figure_S5A_source-image-3-HE.tif]

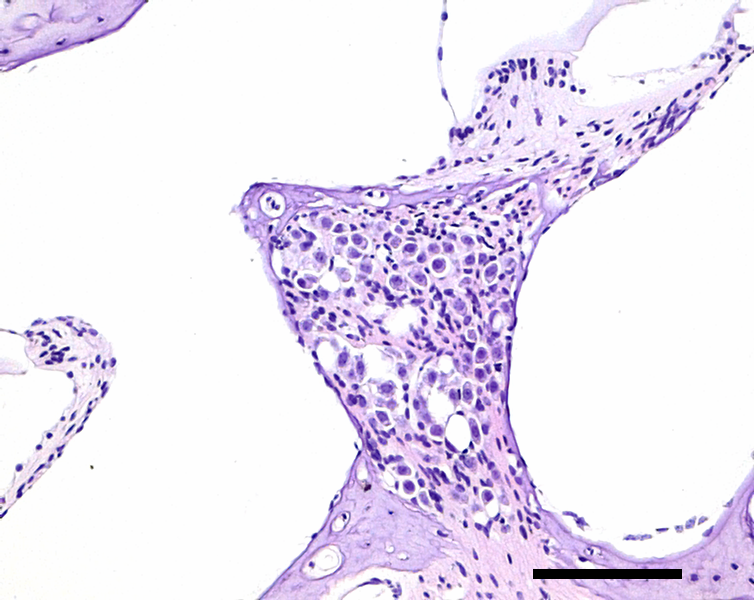

Supplement: Supplementary file 12 — Source Data Appendix Figure S5 [file 44321_2025_350_MOESM12_ESM.zip › Source_Data_Appendix_Figure_S5/Appendix_Figure_S5A_source-image-4-HE.tif]

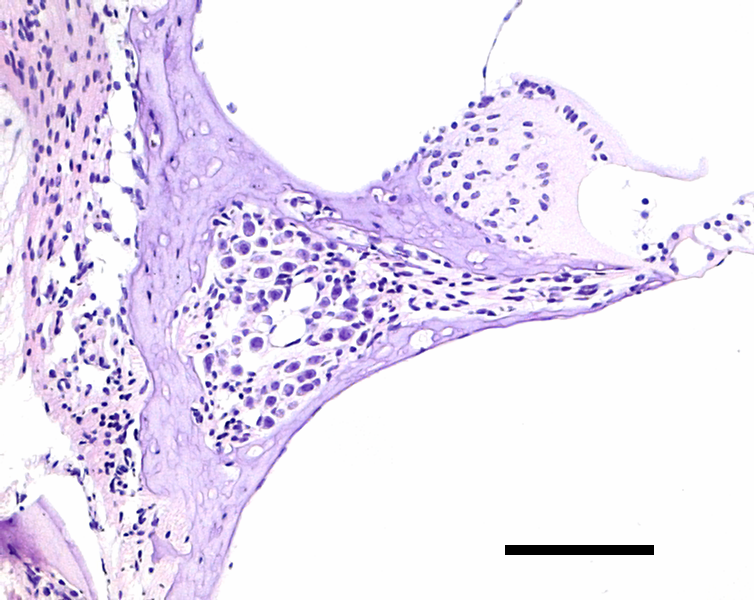

Supplement: Supplementary file 12 — Source Data Appendix Figure S5 [file 44321_2025_350_MOESM12_ESM.zip › Source_Data_Appendix_Figure_S5/Appendix_Figure_S5A_source-image-5-HE.tif]

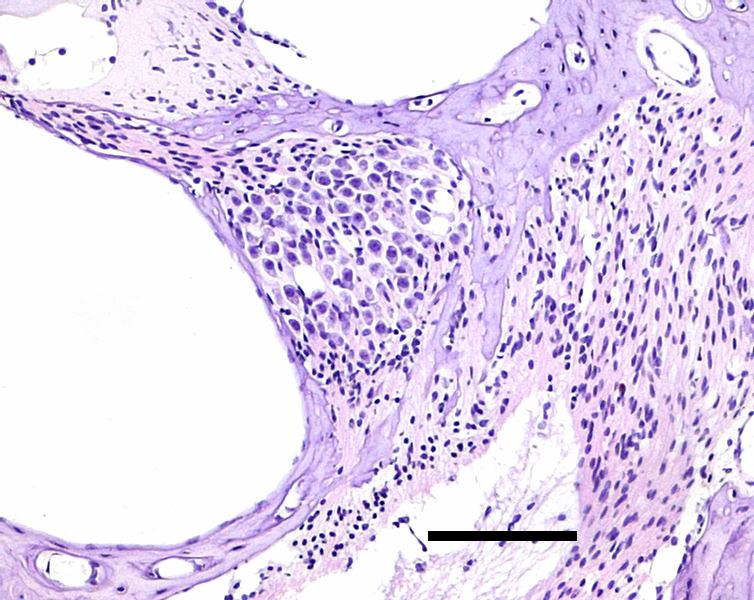

Supplement: Supplementary file 12 — Source Data Appendix Figure S5 [file 44321_2025_350_MOESM12_ESM.zip › Source_Data_Appendix_Figure_S5/Appendix_Figure_S5A_source-image-6-HE.tif]

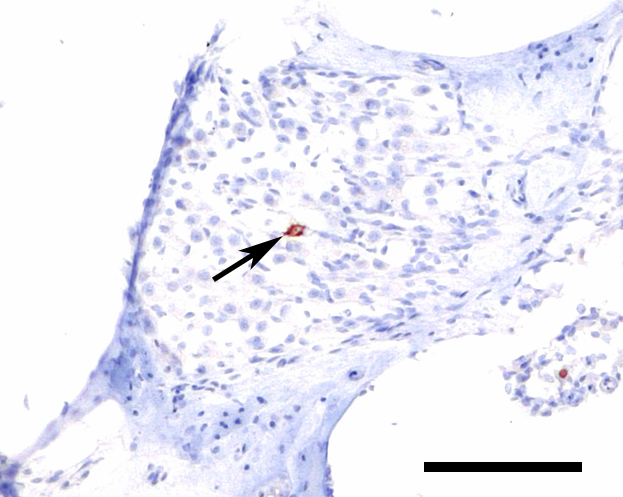

Supplement: Supplementary file 12 — Source Data Appendix Figure S5 [file 44321_2025_350_MOESM12_ESM.zip › Source_Data_Appendix_Figure_S5/Appendix_Figure_S5C_Source-Image-CD3.tif]

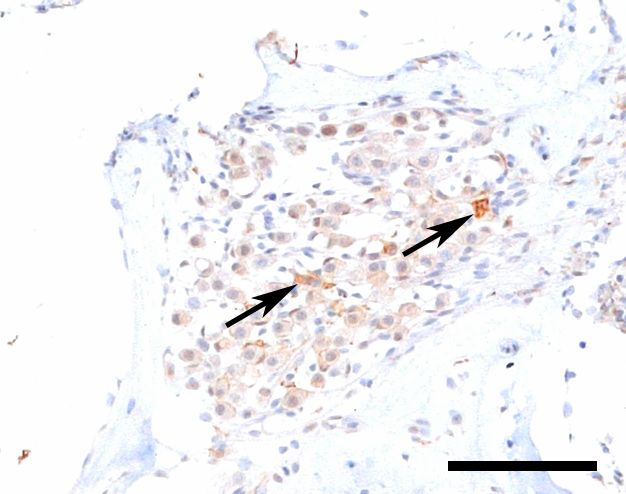

Supplement: Supplementary file 12 — Source Data Appendix Figure S5 [file 44321_2025_350_MOESM12_ESM.zip › Source_Data_Appendix_Figure_S5/Appendix_Figure_S5D_Source-Image-Iba1.tif]
