# Supplementary material for: Channelrhodopsin variants for high-rate optogenetic neurostimulation at low light intensities
Source: EMBO Mol Med. 2025 Dec 9;18(2):462–91. doi: 10.1038/s44321-025-00350-z (PMC12905302; doi:10.1038/s44321-025-00350-z)
Supplement: Supplementary file 13 — Expanded View Figures [file 44321_2025_350_MOESM13_ESM.pdf]

Expanded View Figures

Figure EV1. Photocurrent measurements of blue light-activated ChRs at physiological temperature.

(**A**) Exemplary peak-normalized photocurrents from whole-cell patch-clamp measurements at ~34 °C in NG108-15 cells. ChRs were activated by 50 light pulses of 1 ms at 488 nm (~40 mW/mm^2) at different frequencies (5, 50, 125, and 500 Hz). The lower panels are magnifications of the 500 Hz traces showing photocurrent fluctuations at the stationary state. (**B**) Dependence of the stationary/peak ratio on light pulse frequency. Error bars depict SD. (**C**) Quantification of photocurrent fluctuations normalized to the stationary current amplitude. In panels (**B**, **C**): CatCh: $n = 5$, f-Chronos: $n = 3$, Chronos LC: $n = 3$, f-ChR2 TC: $n = 4$. Error bars depict SD. Statistical comparisons can be found in Table EV3. (**D**) Exemplary peak normalized photocurrents measured in NG cells expressing f-Chronos, Chronos, f-ChR2 TC, or CatCh showing the relation between photocurrent decay kinetics and the transferred charge (area under the curve shown in yellow).

 © The Author(s)

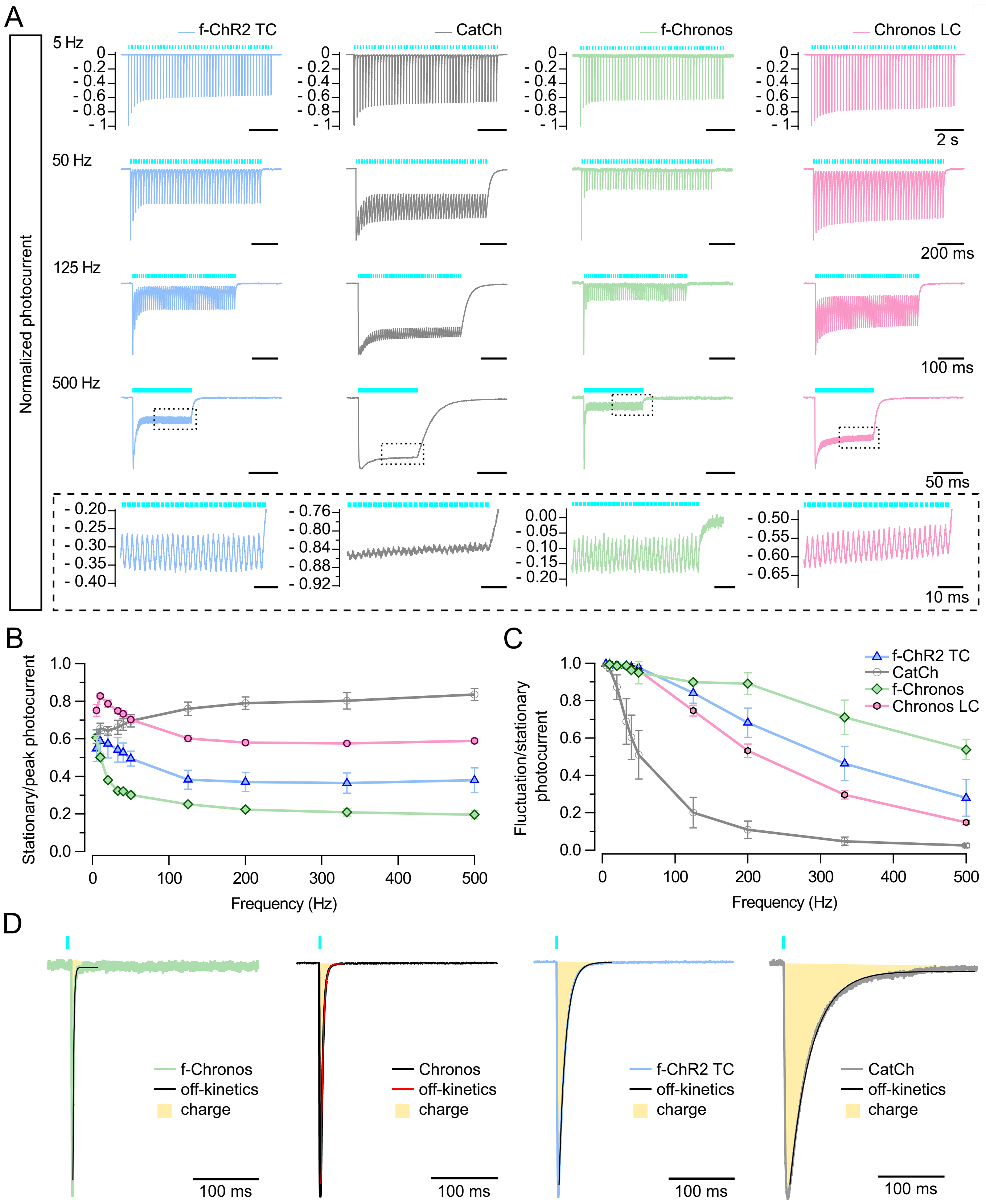
A
f-ChR2 TC
CatCh
f-Chronos
Chronos LC
5 Hz
50 Hz
125 Hz
500 Hz
Normalized photocurrent
2 s
200 ms
100 ms
50 ms
10 ms
B
Stationary/peak photocurrent
Frequency (Hz)
C
Fluctuation/stationary photocurrent
Frequency (Hz)
f-ChR2 TC
CatCh
f-Chronos
Chronos LC
D
f-Chronos
off-kinetics
charge
Chronos
off-kinetics
charge
f-ChR2 TC
off-kinetics
charge
CatCh
off-kinetics
charge
100 ms

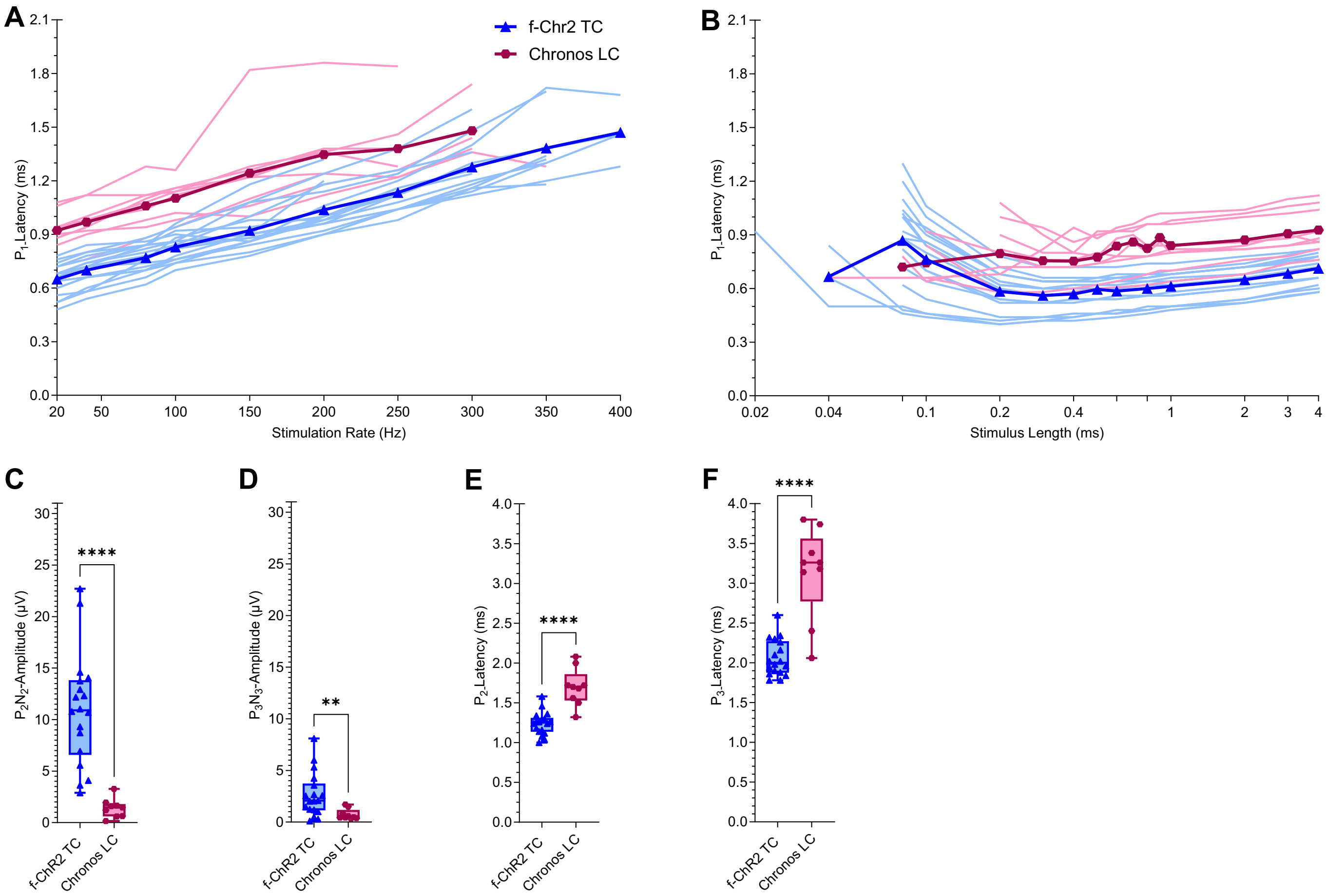

Figure EV2. Analysis of auditory pathway activity evoked by photostimulation of SGNs expressing optimized blue-light-sensitive ChRs.

(**A**) P1 latency of oABRs at varying repetition rate using 1 ms pulses at ~38 to 45.6 mW (bold: mean; faint: all measurements), $n = 18$ mice for f-ChR2 TC, $n = 9$ mice for Chronos LC. (**B**) P1 latency of oABRs for varying pulse durations using ~38 to 45.6 mW pulses at 10 Hz (bold: mean; faint: all measurements) for $n = 17$ mice for f-ChR2 TC, $n = 9$ mice for Chronos LC. (**C–F**) P2-N2, P3-N3 amplitudes and P2, P3 latencies of oABRs for $n = 18$ mice for f-ChR2 TC, $n = 9$ mice for Chronos LC depicting activation of the auditory pathway using ~38 to 45.6 mW, 1 ms pulses at 10 Hz. Data were analyzed as mean ± SD. Center lines represent median values. Boxes show the 25th and 75th percentile and error bars depict minima and maxima. ****$p = 8.53\text{e-}7$ (**C**), $p = 1.09\text{e-}5$ (**E**), $p = 2.62\text{e-}5$ (**F**); **$p = 0.0075$ by two-tailed Mann–Whitney test.

 © The Author(s)

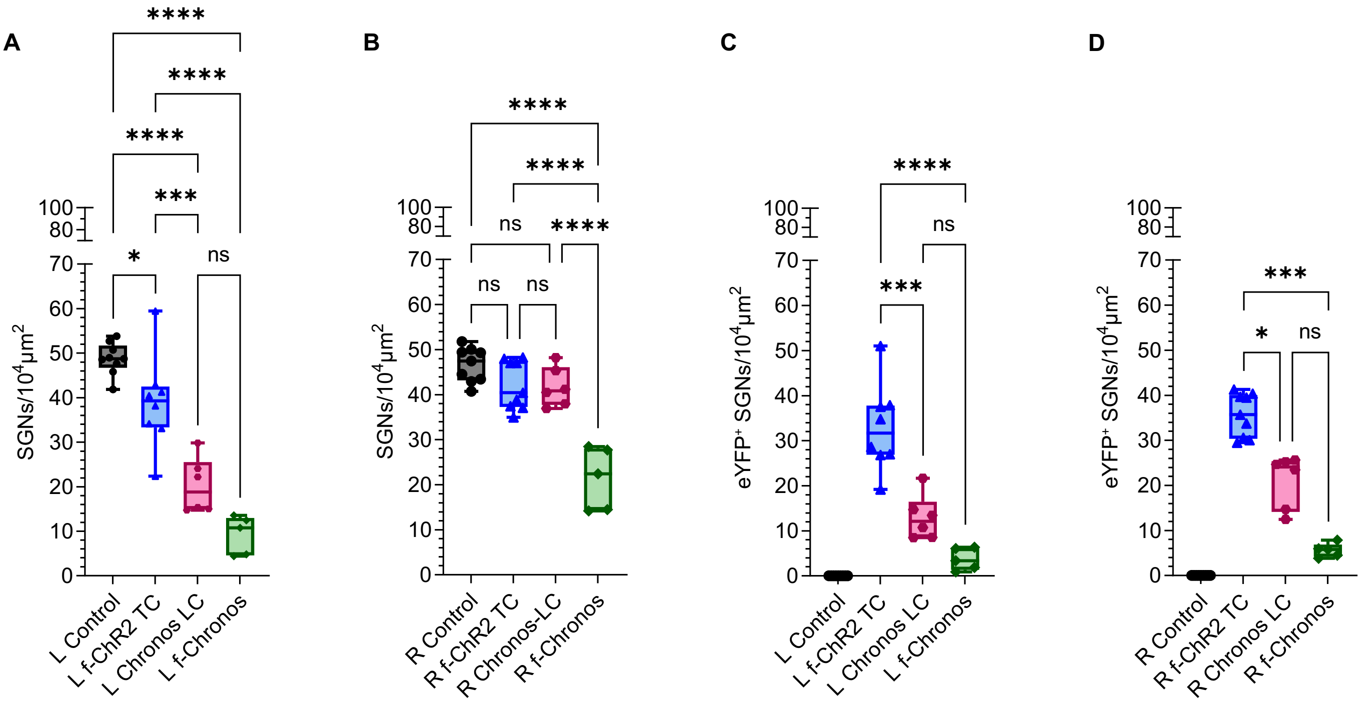

Figure EV3. Immunohistochemical quantification of SGNs expressing optimized blue-light sensitive ChRs.

(**A–D**) Box plots show statistics for the SGN density of left (injected, **A**) and right (non-injected, **B**) cochleae across all cochlear turns, as well as for the density of ChR-expressing (EYFP-positive) cells for the left (**C**) and the right (**D**) side. Quantification includes f-ChR2 TC (blue; $n = 8$ for the left and $n = 9$ for the right cochleae), Chronos LC (violet; $n = 6$ for left and right cochlea), f-Chronos (green; $n = 5$ for left and right cochlea), and non-treated wild-type cochleae (black; $n = 9$ cochlea for both sides each). Center lines represent median values. Boxes show the 25th and 75th percentile and error bars depict minima and maxima. ****$p = 2.68e-7$ (**A**: L Control vs L Chronos LC), $p = 1.74e-9$ (**A**: L Control vs. L f-Chronos), $p = 5.00e-7$ (**A**: f-ChR2 TC vs. L f-Chronos), $p = 1.68e-8$ (**B**: R Control vs. R f-Chronos), $p = 6.14e-7$ (**B**: R f-ChR2 TC vs. R f-Chronos), $p = 3.53e-6$ (**B**: R Chronos LC vs. R f-Chronos), $p = 6.06e-6$ (**C**: L f-ChR2 TC vs. L f-Chronos); ***$p = 0.0002$ (**A**), $p = 0.0003$ (**C**), $p = 0.0002$ (**D**); *$p = 0.0458$ (**A**), $p = 0.0485$ (**D**) by ordinary one-way ANOVA corrected with Bonferroni's (**A–C**) and Kruskal–Wallis test corrected for multiple comparison with Dunn's (**D**).

© The Author(s)

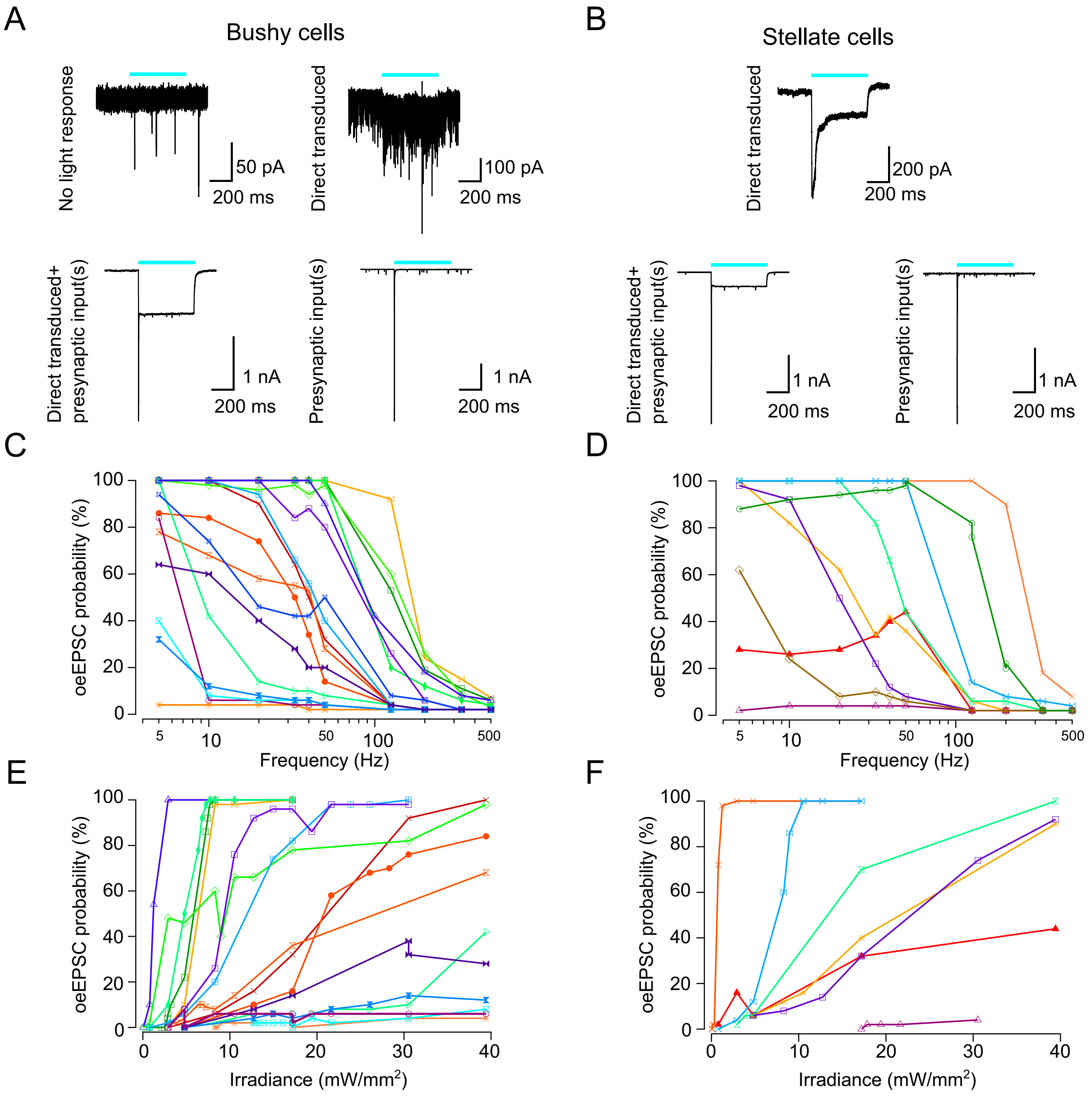

Figure EV4. Variability of photoresponses in principal cells of the AVCN.

(**A**, **B**), Recordings upon long light stimulation (500 ms, 488 nm, ~40 mW/mm²) of either bushy (**A**) or stellate (**B**) cells, indicating transduced principal cells (directly transduced) and non-transduced SGNs, a combination of directly transduced principal cells + transduced SGN presynaptic inputs), or none of them (no light response). Dependence of oeEPSC probability on the stimulation frequency ((**C**), for bushy cells; (**D**), for stellate cells) and the irradiance ((**E**), for bushy cells; (**F**), for stellate cells) in principal cells only receiving presynaptic input(s).

 © The Author(s)
